# Bioaccumulation of Heavy Metals (17 Elements) in the Liver and Kidneys of the Least Weasel (*Mustela nivalis* L.) from Agricultural Areas of Central Europe

**DOI:** 10.3390/toxics14020118

**Published:** 2026-01-27

**Authors:** Gábor Vass, László Könyves, Balázs Berlinger, István Fekete, Attila Bende

**Affiliations:** 1 Institute of Wildlife Biology and Management, University of Sopron, Bajcsy-Zs. u. 4, H-9400 Sopron, Hungary; 2 Department of Animal Hygiene, Herd Health and Mobile Clinic, University of Veterinary Medicine Budapest, István u. 2, H-1078 Budapest, Hungary; 3 Amity Institute of Psychology & Allied Sciences, Amity University Kolkata, Major Arterial Road, Action Area II, Rajarhat, New Town, Kolkata 700156, India

**Keywords:** least weasel, heavy metals, toxicity, liver, kidney, predator mammals

## Abstract

In this study, we investigated the bioaccumulation of 17 heavy metals—titanium, vanadium, chromium, manganese, iron, cobalt, nickel, copper, zinc, arsenic, selenium, molybdenum, antimony, cadmium, tin, mercury, and lead—in the liver and kidney tissues of the least weasel, based on samples (n = 129) collected from adjacent intensive agricultural environments in Hungary and Austria. To explore the structure of the bioaccumulation data, principal component analysis (PCA) was performed. The PCA score plot based on national-level elemental profiles revealed no differentiation between Austria and Hungary. In contrast, a clear and unambiguous distinction was observed between the two examined tissues within individuals for Ti, Mn, Fe, Co, Zn, Se, Mo, Cd, and Hg (*p* < 0.001), as well as for Pb (*p* < 0.05). The biological relevance of the accumulation results was adjusted using the MCID approach. As heavy metal accumulation in the least weasel has not yet been investigated, our results could only be compared with concentrations reported for predatory mammals occurring in similar habitats. Based on the relevant literature, we highlight predominantly anthropogenic exposure pathways affecting agroecosystems—organic and mineral fertilizers, plant protection products, wastewater, and fossil fuels—which underscore the necessity of regular biomonitoring studies in agricultural landscapes.

## 1. Introduction

Heavy metals are defined as substances with a density greater than 4–5 g/cm^−3^ [1,2,3] that enter the environment from natural and anthropogenic sources. Natural heavy metal concentrations can be attributed to volcanic activity, erosion of ore-bearing rocks, and atmospheric deposition, but numerous human activities—mining, use of pesticides and fertilizers, burning of fossil fuels, etc.—also contribute to the accumulation of these materials [4,5,6,7,8]. Several studies drew attention to the fact that the accumulation of heavy metals had a negative impact on natural ecosystems, contributing to a decline in biodiversity [9,10]. Furthermore, due to their toxicity, they deserve close attention from a public health perspective [4,5,11]. Accordingly, in recent years, research aimed at detecting heavy metal content has become increasingly prevalent in ecotoxicological studies, with a strong emphasis on mapping and monitoring bioindicator species. In this context, wild mammals represent an important group of organisms, but several factors must be taken into account when selecting bioindicator or bioenvironmental indicator species [12]. For example, terrestrial carnivorous mammals can be considered biomonitor organisms based on their position in the trophic levels [13,14,15,16]. In addition, an important aspect is the assessment of herbicide exposure in an intensively managed agricultural environment similar to our study area, which allows us to gain insight into the heavy metal burden of a small predator at an intermediate trophic level. Heavy metal content tests were carried out on several species of mustelidae, including the Eurasian otter (*Lutra lutra*) [17,18], the Beech marten (*Martes foina*), the Martes (*Martes martes*) [18,19], the American mink (*Mustela vison*) [18,20], the European polecat (*Mustela putorius*) [18,21], and the European badger (*Meles meles*) [18,22,23]. However, heavy metal accumulation testing has not yet been conducted on the least weasel, despite the fact that, based on the limited number of studies on habitat use and feeding ecology, this species may be significantly exposed to heavy metal contamination. The habitat of the least weasel extends beyond intensive agricultural habitats to populated areas [24,25], exposing it directly and indirectly to anthropogenic heavy metal pollution. Based on the results of dietary biology studies, small rodents are the primary dietary source for this species [26,27,28], but arthropods are also included in its diet composition [28,29,30], so we can assume that it is exposed to heavy metals from rodenticides and insecticides. In carnivorous mammals, lead shots from hunting can also be an important route of exposure to toxic heavy metals [31]. However, in predatory mammals, a significant exposure route to toxic heavy metals can be lead from hunting ammunition [31]. However, an even more important source of contamination may be the heavy metal load resulting from the use of fossil fuels by agricultural machinery [32], while the most pronounced emission factor is the herbicide load associated with the intensive management of agricultural crops [8,33,34,35]. The selection of our study sites and the broad-spectrum analysis of heavy metals were justified precisely by this latter factor. In our study, we evaluated the accumulation of 17 heavy metals (Ti, V, Cr, Mn, Fe, Co, Ni, Cu, Zn, As, Se, Mo, Sb, Cd, Sn, Hg, and Pb) based on the two most commonly studied tissues (liver and kidney) in ecotoxicology, known as sites of accumulation of substances in significant concentrations and responsible for detoxification [36]. Small predators at intermediate trophic levels can be important biomonitoring organisms in such studies, and our results can provide important guidance for determining normal and toxic thresholds for heavy metals in mustelidae. Accordingly, the main aim of our study is to understand the characteristics of heavy metal accumulation (liver/kidney ratios, priority metal profiles), representing a significant contribution based on primary data to the ecotoxicological knowledge of this understudied small carnivore.

## 2. Material and Method

### 2.1. Field Sampling

The samples were collected in agricultural environments in Hungary and Austria. The least weasel was partially protected in Hungary in 1974, and then, with the abolition of partial culling, it has enjoyed full protection since 1988 [37], so our sample collection was carried out with the permission of the nature conservation authority (GY/41/01351-7/2023). In Austria, this small furry predator is not protected in the province of Burgenland, where the sample collection took place, and can even be hunted all year round [38,39], but its import into Hungary is subject to a permit (GY/41/01351-7/2023). The device used for sample collection—in accordance with the nature conservation authority’s permit—is a Fenn Mark 4 spring trap for killing, which meets the requirements for legal trapping devices set out in Section 36 (1) of Act CLXXXIII of 2015. The Hungarian sample collection site is located on the special-purpose industrial site of Lajta-Hanság Zrt. on the Moson Plain (Mosoni-sikság) (N 47°47′33″–47°52′18” E 17°03′37″–17°09′50″). The Hungarian samples (n = 51) were collected between 20 August 2023 and 14 August 2024, while the Austrian specimens (n = 14) were collected in 2023. The Austrian sampling site was in the Nickelsdorf municipality (N 47°56′55″–47°56′31″ E 17°03′01″–17°02′57″). Both the Hungarian and Austrian sampling fields are intensively managed agricultural landscapes, where woody vegetation occurs mainly along field edges and is generally limited in extent. There are no industrial facilities nearby, and the transport network consists of secondary roads with low traffic; therefore, the primary potential source of heavy metals is the use of the intensive agricultural environment.

It is justified to evaluate the two sampling areas separately, as the straight-line distance between them is approximately 22 km, which lies far beyond the average home range of the least weasel reported in the literature ([40,41]; maximum 26.3 ha). In addition, the sufficiently large distance implies that the two areas may have been exposed to different heavy metal loads, and the long-term accumulation of these toxic compounds is a well-documented phenomenon [42]. Furthermore, it must be taken into account that the range of permitted plant protection products, fungicides, and insecticides differs partly between Austria and Hungary, which may result in differences in heavy metal exposure between the Austrian and Hungarian sampling areas. Although both Hungary and Austria apply the same EU regulation (Regulation (EC) No 1107/2009), national authorization documents may impose additional bans or restrictions on certain active substances. For example, glyphosate—although approved at the EU level—is restricted under Austrian national regulations for private use and in sensitive areas. This also applies to widely used fungicide components such as copper hydroxide and copper oxide compounds. The Fenn Mark 4 traps used were installed in a closed box, a so-called “tunnel trap,” with the aim of ensuring selectivity for the target species; therefore, unwanted species—especially coexisting protected predator species—were not caught as a result [43]. During trapping, a pronounced sex imbalance favoring males was observed, consistent with international literature [44,45]. Since only a single female was captured, her data were excluded from the analysis. According to the literature, the average age of trapped individuals is 0.79–1.16 years (n = 455) [44]; therefore, age cannot be considered an influencing factor in accumulation for these short-lived animals. For this reason, no precise age determination was performed.

### 2.2. Analytical Tests

We examined a total of 129 samples (64 liver and 65 kidney) for the accumulation of 17 heavy metals (titanium (Ti), vanadium (V), chromium (Cr), manganese (Mn), iron (Fe), cobalt (Co), nickel (Ni), copper (Cu), zinc (Zn), arsenic (As), selenium (Se), molybdenum (Mo), antimony (Sb), cadmium (Cd), tin (Sn), mercury (Hg), and lead (Pb)). Reagents suitable for trace analysis were used for sample preparation. Hydrogen peroxide (30 m/m%, AnalaR NORMAPUR), nitric acid (69 m/m%, Aristar), hydrochloric acid (37 m/m%, Aristar), and ethanol (AnalaR NORMAPUR) were purchased from VWR International Ltd. (Leicestershire, UK). Deionized water (18.2 MΩ/cm) was prepared using a Purite Select Fusion 160 BP water purification system (SUEZ Water Technologies & Solutions, Trevose, PA, USA). For the calibration of the inductively coupled plasma mass spectrometer (ICP-MS), we used a multi-element standard solution (Instrument Calibration Standard 2, TruQms); internal standard elements were added to the samples, calibration solutions, and blank solutions in the form of Internal Standard Mix (TruQms) solution purchased from Perkin Elmer Inc. (Waltham, MA, USA). For quality control, we used a certified reference material (1577c Bovine Liver) purchased from Merck KGaA (Darmstadt, Germany). The argon gas used was 4.8 purity and was purchased from Messer Hungarogaz Kft (Budapest, Hungary). During sample preparation, 0.5 g of each sample was weighed into CEM MARS XPreSS Teflon vessels, 5 mL of hydrogen peroxide and 5 mL of nitric acid were added, and the samples were digested in a CEM MARS6 microwave digestion system (CEM Corporation, Matthews, NC, USA) under the following parameters: 35 min heating to 200 °C; 50 min holding; MW power 1700 W; 40 positions. After digestion, the contents of the Teflon vessels were transferred to 50 mL polypropylene (PP) tubes (Deltalab, Rubí Barcelona, Spain). The solutions were then filled up to 25 mL with deionized water and diluted 5-fold with deionized water in 12 mL PP tubes (Deltalab, Rubí Barcelona, Spain) prior to analysis, after adding 0.4 mL of ethanol and 100 µL of internal standard solution. The internal standard solution contained 1 µg/mL of bismuth (Bi), germanium (Ge), and indium (In). The samples for quality control and the blank samples were prepared in the same way. Between digestion cycles, the Teflon vessels were cleaned with 0.15 M hydrochloric acid solution. The elements were determined using a Perkin Elmer NEXION 2000 (Perkin Elmer, Waltham, MA, USA) ICP-MS in helium KED mode (the cell gas flow rate was 4.8 and 5.8 mL/min, depending on the element measured). The following instrument parameters were used during the measurement: solid-state RF generator, 34.5 MHz LumiCoil; RF power: 1600 W; nebulizer: Meinhard plus quartz, small internal volume, type C; plasma gas flow rate: 15 L/min; auxiliary gas flow rate: 1.2 L/min; nebulizer gas flow rate: 1.06 L/min.

Table 1 summarizes the monitored isotopes, applied internal standards, limits of detection (LoD), and quality control results obtained using the certified reference material SRM 1577c (bovine liver). For each element, certified values, measured concentrations, and recoveries are reported where applicable. When certified values were unavailable or measured concentrations were below the respective LoD, quality control was assessed using spiked samples.

### 2.3. Statistical Methods

Statistical analyses were carried out in RStudio (version 4.3.1) [46]. No imputation was performed, as there were no missing data. Raw LoD (Limit of Detection) values were retained in their original form to ensure methodological rigor and data transparency. The original laboratory numbers were taken. First, standard descriptive statistics were computed, and then normality statistics using the Shapiro–Wilk and Kolmogorov–Smirnov tests. The dependent variables were 17 heavy metals. The independent variables were organ type (within-individual variable: kidney and liver) and country (between-individual variable: Austria and Hungary). The between-country comparison was performed for Austria and Hungary. Because the same individuals have measurements for both liver and kidney tissue, the liver-kidney comparison is a within-subject design.

Two Principal Component Analyses (PCA) were performed on the correlation matrix (rather than the covariance matrix) via z-standardization without any rotation in order to perform variance decomposition and structure discovery rather than latent factor interpretation. First, we conducted a PCA on the row-wise observations of bioaccumulated metals that were measured in pairs of organs, and then another PCA to explore if the two countries (Austria and Hungary) can be separated in the analysis. Only numeric variables for metal concentration were used. Before extraction, all variables were z-standardized to avoid the dominance of components due to scale disparities among elements. The 17 dependent variables entered into the PCA were all the 17 continuous bioaccumulation variables. A score plot was made to visualize the results. The organ type was not included in the PCA, neither country in the second PCA; organ type and country were utilized solely for interpretation. We were primarily interested in the multivariate separation of the two organs and the two countries, respectively. Membership in an organ was determined by “Sample Code”, which was a variable in the dataset. Codes that start with “L” stand for liver samples, and codes that start with “K” stand for kidney samples.

Eigenvalue decomposition of the correlation matrix was used to extract components, which is the same as the singular value decomposition of the standardized data matrix. There was no limit on the number of components that could be extracted. Eigenvalues and cumulative explained variance were used to figure out how much each component explained the variance. The first two components were the most important because they explained the most variance and were important for organ differentiation.

Rotation was deliberately not used as the main aim of the analysis was dimension reduction and spatial separation of observations, rather than the identification of interpretable latent constructs. Unrotated principal components retain the maximal variance characteristic and uphold orthogonality, which is crucial when component scores are later employed for visualization and group differentiation (e.g., kidney versus liver). Using orthogonal or oblique rotation, usually justified in factor analysis, would have changed the component loadings without making the variance capture better, and it would have made it harder to understand the geometric meaning of the component scores.

Because we were interested in which trace elements contributed most to the separation, we looked at the size and sign of the component loadings to figure out what they indicated. Loadings of about |0.35| or higher were considered to be substantively meaningful in biomedical and environmental research, in line with conventional PCA practice [47]. Using the standard regression method (i.e., linear combinations of standardized variables weighted by component loadings), we figured out the component scores for each observation. After that, these scores were used to make two-dimensional score plots (PC1 vs. PC2) and convex hull representations of organ groups (colored in orange and blue).

The PCA did not include organ type (kidney vs. liver) in the analysis. Instead, organ membership was determined post hoc from the variable “Sample Code”, where labels that started with “K” indicated kidney samples and labels that started with “L” indicated liver samples. The only things that used the organ type were convex hull construction and graphical annotation for interpretative purposes. There were no random elements in the PCA itself.

Shapiro–Wilk and Kolmogorov–Smirnov tests were conducted (liver–kidney) to assess the normality assumption. If the *p*-value of the Shapiro test was 0 < 0.05, normality was rejected, then the Wilcoxon signed-rank test was employed. Otherwise, if normality held, the paired-samples *t*-test was conducted. The test statistics (W) with the *p*-values from the Shapiro–Wilk tests and the uncorrected *p*-value from the parametric or non-parametric tests are not reported for easier reading. Benjamini–Hochberg adjustment (BH) [48] is used to control the False Discovery Rate (FDR).

Half a standard deviation MCID (Minimal Clinically Important Difference) is used to filter out the biologically irrelevant differences [49,50]. Formally, if the mean and standard deviation of a variable (e.g., bioaccumulation or biomarker level) are denoted by μ and σ, respectively, then any observed difference ΔX is filtered using the criterion: ΔX ≥ 0.5σ. In other words, only those differences that exceed this threshold are interpreted as minimal biologically important. All smaller differences are filtered out as likely biologically irrelevant.

Particularly in biomedical or ecological research, the 0.5 SD rule can help identify biologically meaningful deviations in parameters such as blood markers, pollutant levels, or population sizes, avoiding the overinterpretation of trivial but statistically significant changes [49]. The 0.5 SD metric is rooted in the work by [50], who demonstrated across multiple clinical and psychological outcome measures that a change in half an SD approximated a meaningful difference. Hence, after applying the BH-adjustment, we used the MCID metric as a threshold to distinguish differences that are likely to be biologically meaningful from those that are merely statistically significant but trivial in a real-world biological context.

We contend that the epistemological basis of MCID can be transferred to toxicological domains too (for a review of the transferability in biological domains, see [51]). The problem with the reliance on null-hypothesis significance testing in toxicology is that in large datasets or biomonitoring studies, toxicologically trivial (i.e., small) effects can be amplified statistically, that is, giving rise to statistically false positive results. Hence, MCID is not just confined to symptom change or clinical scales. Rather, it is a generalizable framework that is applicable whenever decisions are contingent on the magnitude of an effect.

The structure of the present study design determines how to calculate a 0.5 SD threshold for the MCID. For example, when comparing independent groups (like different populations or experimental conditions; in our case, Austria and Hungary) in between-subject designs, the pooled standard deviation is usually used to set the 0.5 SD criterion. This pooled value takes into account the differences between the two groups and gives us one estimate of how much the mean difference varies. This method finds the MCID by taking half of the pooled SD. This has been shown in many real-world situations to be close to a threshold for meaningful change or group-level distinction. A lot of research and theory from fields like clinical medicine, psychology, and health outcomes research support this approach [50,52,53].

However, when working with within-subject data (also called paired or correlated data), where the same individuals are measured (i.e., kidney and liver), the right approach is to find the SD of the change scores instead of the raw score variability. This method captures the differences between individuals and is more in line with the nature of paired designs. The MCID is then defined as half of the SD of these change scores for each individual. This method is usually applied for longitudinal studies and clinical trials, which measure how sensitive individuals are to change, and is the main goal [54,55]. However, as we noted earlier, we adopted the MCID framework to filter out toxicologically irrelevant effects. From a methodological point of view, both pre-and post-trials and within-individual designs are treated the same way statistically: Liver data and Kidney data are correlated data, as the two bioaccumulation values belong to the same animal. Also, the two organs are literally within the same specimen.

Most recently, ref. [56] made it clear again that a within-subjects (repeated-measures) design can be conceived of as “even multiple simultaneous measurements from different parts of the same subject”, demonstrating the second interpretation of within-subject designs ([56]; see, repeated measures design definition, 2025). Let us cite [56], “Repeated measurements can also be taken under different experimental conditions; furthermore, measurements can be taken from the same subject at a single point in time. This could involve observations from various parts within a single subject”. Lastly, MANOVA might come to mind as a way of analyzing the data. Importantly, MANOVA assumes multivariate normality of the dependent variables and homogeneity of covariance matrices across groups [47,57]. Therefore, we did not run MANOVA, given that most of the dependent variables violated multivariate normality severely. Ref. [47] explicitly claims that violations of multivariate normality are a concern in MANOVA in small samples like the one in our study. Also, MANOVA does not tell how many of them are important, or whether the differences are toxicologically trivial or meaningful. Instead of MANOVA, we ran PCA on the kidney and the liver data.

### 2.4. Discussion Concept

We are not aware of any studies on the tissue accumulation of heavy metals in the least weasel; only one Italian study reports data on the Pb load of this small carnivore [58]. Accordingly, in our study, we compared the quantitative values of heavy metal concentrations detected in the liver and kidney tissues of the least weasel with the results of international studies that examined heavy metal accumulation in the tissues of terrestrial carnivorous mammals whose habitat and potential dietary base are largely similar to those of the least weasel. These factors are important considerations when interpreting heavy metal accumulation due to exposure and biomagnification characteristics [59]. The habitat selection and habitat use of terrestrial related species can be influenced by a number of factors, but the occurrence of European polecat, beech marten, pine marten, and canine mesopredators (red fox, golden jackal) in habitats preferred by the least weasel is a well-known phenomenon [60,61,62]. In terms of their dietary strategy, these species are less specialized than the least weasel, but small mammals make up a significant part of the diet of all of these predators. Small mammals often dominate the diet of the European polecat (*Mustela putorius*) (rodents 69%—[63]; 51.7%—[64]). In addition to fruit, birds, and arthropods, small mammals are an important component of the diet of the beech marten (*Martes foina*) (36.1%—[65]), and small mammals may also constitute a significant proportion of the diet of the pine marten (European review 42–47% [66]; 26.6% [67]). Among related species, the consumption of small mammals may play a seasonally important role in the diet of larger-bodied badgers [68], as is the case with the red fox [60,69] and the golden jackal [69,70,71]. The quantitative data for heavy metals are given in terms of wet weight. To ensure comparability of the heavy metal data measured in our study with literature values, the metal concentrations were converted to wet weight in all cases, using the formula provided by [72]. To express the moisture content of different tissue types, we used the average values described by [73] (muscle—74.5%, liver—72.1%, kidney—74.8%) as a basis. In the studies used as a basis for comparison, we specifically took the average concentration values of the metal content of tissue types identical to those in our study, as the bioaccumulation value of the metals examined may vary depending on the tissue type [74].

## 3. Results

First, we are reporting on descriptive statistics for each heavy metal grouped by organ type and then by country. Then, we want to explore if there are any differences in the bioaccumulation of heavy metals between the liver and the kidney, regardless of country. Second, we are interested in potential differences between Austria and Hungary regarding the bioaccumulation of heavy metals.

Table 2 shows the average concentrations of the 17 heavy metals tested in the liver and kidneys of weasels, regardless of the location where the samples were collected.

The quantitative distribution of heavy metals in the soft tissues of the least weasel showed the following pattern: in liver samples: Fe > Zn > Cu > Mn > Mo > Se > Cr > Ti > Ni > Hg > Sn = Pb > Co > Cd > V = As = Sb; in kidney samples: Fe > Zn > Cu > Se > Mn > Cr > Mo > Ti > Ni = Hg > Cd = Pb > Sn = Co > V > As = Sb.

Assessment of distributional assumptions revealed substantial departures from univariate normality for nearly all dependent variables. Shapiro–Wilk and Kolmogorov–Smirnov tests were statistically significant for the majority of variables. Shapiro–Wilk tests indicated that 16 out of 17 numerical variables (94.12%) violated normality highly significantly (*p* < 0.05), with only cobalt showing an approximately Gaussian distribution (W = 0.980, *p* = 0.053). Given that the multivariate normality assumption is strongly violated, the routine use of classical MANOVA, Pearson correlations, or Gaussian linear models is contraindicated [47]. Yet, it justifies the use of PCA based on the correlation matrix (which is robust to non-normality).

Based on a comparison of the concentrations of 17 heavy metals in liver and kidney samples, the average values for Ti, Mn, Fe, Ni, Zn, and Mo were higher in liver tissue, while higher cumulative average values were found in kidney tissue for V, Cr, Co, Cu, Se, Cd, Hg, and Pb. The average concentrations of As, Sb, and Sn were similar in the tissue types examined (Table 2). Accordingly, we found significant differences between the two tissue types in the average values of Ti, Mn, Fe, Co, Zn, Se, Mo, Cd, Hg (*p* < 0.001), and Pb (*p* < 0.05) (Table 3). After applying the MCID (0.5 SD) criterion, Hg and Pb were considered biologically irrelevant due to insignificant differences among the ten heavy metals listed above (Table 4).

The first PCA aimed to explore the clusterability of the two organs. The first principal component (PC1) accounted for 39.31% of the variance, while the second component (PC2) accounted for 25.90%. Together, they accounted for 65.22% of the total multivariate variance. This is acceptable for high-dimensional bioaccumulation data. The PCA score plot reveals a clear structure: a dense cluster represents two clearly distinguishable similar samples (coupling with the two organs), while five samples are displaced. This suggests distinct bioaccumulation patterns and not random noise.

From a toxicological standpoint, these outliers are not statistical artifacts but are biologically and toxicologically meaningful. Specifically, PC1 was mainly characterized by loadings for Mn (−0.42), Fe (−0.35), and Co (0.37), indicating a general bioaccumulation gradient that differentiated kidney samples from liver samples. PC2 was mostly made up of Zn (0.59) and Cu (0.52), which shows how the liver controls trace metals. A visual inspection of the component scores showed that kidney and liver samples were systematically separated along PC1. There was also some separation along PC2, which could be explained by liver-specific accumulation patterns.

**Figure 1:**
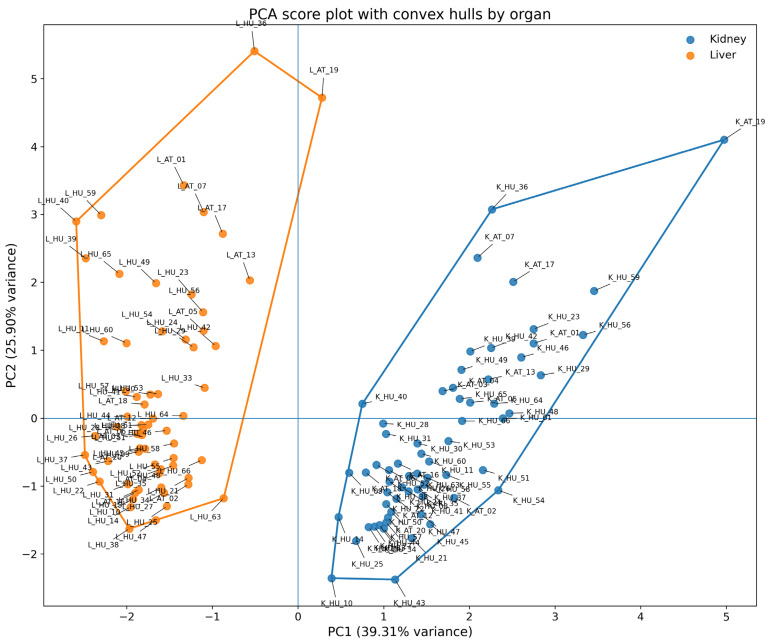
Principal component analysis (PCA) score plot (PC1 vs. PC2) of z-standardized metal concentrations in liver and kidney samples, irrespective of country. The plot illustrates clear organ-specific multivariate bioaccumulation patterns. Note. Samples are colored by organ type (kidney and liver) as derived from Sample Code (labels starting with “L” designate liver data with the dots in orange, while labels starting with “K” designate kidney data with dots in blue). Cases are labeled with full Sample Codes, where “L” designates liver, while “K” designates kidney. Cases are labeled with full Sample Codes, where “L” designates liver, while “K” designates kidney.

The PCA shows that PC1 represents a pattern of global bioaccumulation (Figure 1). Also, PC1 turned out to be the main line that separates samples from the liver and kidney with a 100% accuracy. PC1 has very high negative loadings for iron and manganese, but a positive loading for cobalt. This pattern shows that organ-specific differences cannot be attributed to a single metal. Instead, they show systematic differences between the kidney and the liver. PC2 is an axis of hepatic trace-metal enrichment that shows large positive contributions from zinc and copper and a moderate contribution from manganese.

Furthermore, it is conspicuous upon closer inspection of the plot that the datapoints do not cluster by country, that is, values belonging to Austria and Hungary can be found in both clusters. To support this claim further, another PCA was carried out to examine if liver and Kidney data are clustered like the two organs.

**Figure 2:**
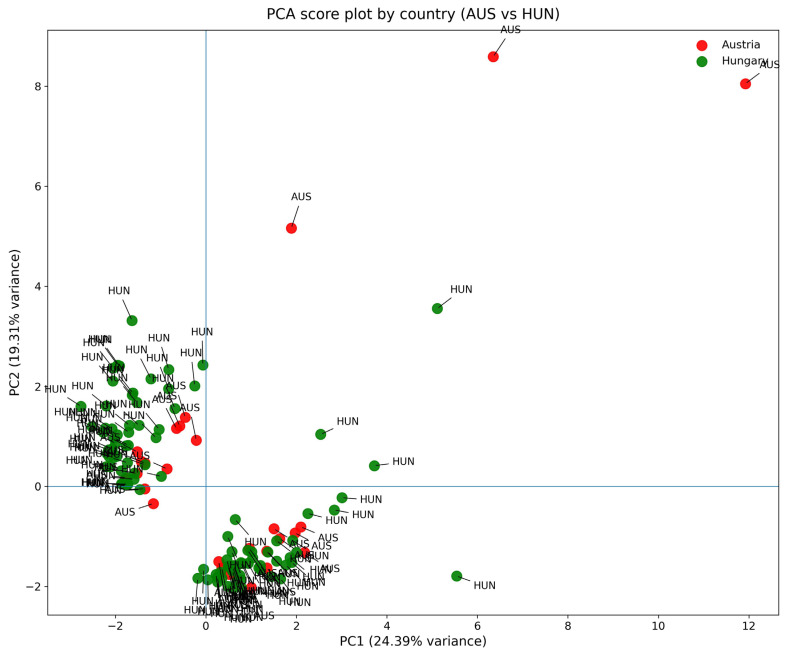
PCA score plot (PC1 vs. PC2) of z-standardized metal concentrations in liver and kidney samples by country. Note. The plot illustrates bioaccumulation data of the liver and the kidney, consistent with Figure 1. However, there is no separation by country in this PCA plot in Figure 2. Site location is color-coded as follows: red dots designate an organ datapoint from Austria (labeled as AUS), while green dots designate one from Hungary (labelled as HUN).

The PCA score plot in Figure 2 with country-level elemental profiles shows a clear and systematic multivariate separation, except between Austria (AUS; red) and Hungary (HUN; green). This separation shows the overall bioaccumulation patterns of the two organs (Figure 1), and not the separation by country, consistent with the non-significant pair-wise comparisons (parametric and non-parametric tests). Figure 2 shows that the two countries are in different parts and clusters of the multivariate element space. Specifically, almost the same number of elements from Austria are in both clusters. Based on the visual inspection of the score plot, we suspected no trace elements to contribute to a separation. Consistent with our conjecture, examination of the unrotated factor loadings indicates that separation along PC1 is not driven by a single dominant metal. Hence, country separation in the PCA plot cannot be attributed to a single or even only two variables.

We examined whether statistically verifiable differences in heavy metal concentrations could be detected in liver and kidney samples from specimens originating from different countries (Table 5 and Table 6). In liver samples, there was a significant difference in Ti (BH Adjusted *p* < 0.05) between Hungarian and Austrian tissue samples (Table 5). An MCID of 0.5 SD criterion was run on Ti in the liver data to see if the observed difference exceeds the MCID threshold. The observed mean difference in Ti levels between Austria and Hungary is 0.07, which does not exceed the MCID threshold of 0.07 (defined as 0.5 times the pooled SD of 0.15). This indicates that the difference in liver Ti accumulation between the two countries is merely statistically detectable but not meaningful in magnitude. Therefore, it can be interpreted as biologically or toxicologically not relevant.

Thus, the PCA score plot based on national-level elemental profiles did not reveal any difference between Austria and Hungary. In contrast, a clearly distinct separation was observed between the two organs. These observations are consistent with both the inferential statistical analyses and the MCID results.

Parametric and non-parametric statistics revealed that there are no differences between the two countries regarding the bioaccumulation of the 17 metals (see Table 6), congruent with the results of the parametric and non-parametric statistics in Table 5.

## 4. Discussion

### 4.1. Accumulation Values of Essential Elements: Comparison of Our Data with Data on Terrestrial Predators

Of the 17 heavy metals we examined, the following elements can be considered essential elements for mammals: Fe, Zn, Cu, Mn, Se, Cr, Co, and Mo [59,75,76,77].

The accumulation of Fe in the tissues of carnivorous mammals is more significant than in herbivorous mammals, which can be attributed to their diet composition, as inorganic Fe compounds present in plants are more difficult to assimilate than those found in an animal-based diet. For this reason, carnivorous mammals are important biomonitoring organisms, yet the tissue accumulation of this essential element is understudied in terrestrial mustelidae. In mammals, Fe accumulates in significant amounts primarily in the liver and spleen, and in smaller concentrations in the kidneys and skeletal muscles [78]. We also confirmed this quantitative variability between tissue types in the least weasel. The Fe concentration described in the liver of canids ranges from 103.87 to 283.81 mg/kg, while the concentrations measured in the kidney are lower, ranging from 44.58 to 64.91 mg/kg (Table S1). Based on our study, the average concentration detected in liver tissue falls within the above ranges, while the nephritic value exceeds the values documented in terrestrial predator species. In our study, the hepatic Fe content of the least weasel exceeded the concentrations measured in red foxes and golden jackal samples from Romania and Hungary. A value identical to our result was published from golden jackal liver samples in Croatia. The nephritic Fe content we determined was, on average, about three times higher than the concentrations published in European studies of these two mesopredators (Table 2 and Table S1). The tissue concentration of Fe varies significantly among different taxa, making it difficult to interpret its role and significance [78]. The least weasel is a small predator with a specialized diet strategy, and small rodents make up a significant part of its diet [28], accounting for up to 95% of its diet intake [29]. In contrast, plant-based diet components are present in higher proportions in the diet of red foxes and golden jackals [70,79,80,81], which may contribute to the quantitative differences in Fe concentration described above. In addition to the nutritional reasons mentioned above, Fe tissue enrichment can also be attributed to habitat and habitat use factors [33,34,78].

Based on the results of the cited studies, Zn bioaccumulation is more significant in liver tissue than in kidney tissue, which is also confirmed by the results of our study. The values ranged from 8.39 to 99.12 mg/kg in liver samples and from 4.28 to 41.32 mg/kg in kidney samples in terrestrial predators, and the concentrations detected in our study were consistent with these values regardless of tissue type (Table 2 and Table S1). The concentration described in the liver tissue of the least weasel is very similar to the concentrations measured in Spain [14,23] and in Hungary in liver samples from red foxes [82]. The hepatic Zn concentration in the least weasel is lower than the values reported for the beech marten and the European pine marten in Italy [19], but in the vast majority of cases, it exceeds the accumulation values reported for canine predator species in both liver and kidney tissue samples [14,31,76,83,84,85,86].

The accumulation of Cu in tissue has been well researched in both small and medium-sized terrestrial predator species. The accumulation of this essential element in liver tissue ranges from 5.97 to 36.1 mg/kg, while concentrations found in kidney tissue range from 2.32 to 18.70 mg/kg (Table S1). The average values measured in both tissue samples from the least weasel fall within this range. Studies that measured Cu accumulation in both liver and kidney samples reported higher values in the liver in most cases. In contrast, in our study, we found a higher concentration of 0.32 mg/kg in the kidneys, which is consistent with the variability between tissue types identified by [15] in wild red foxes (Table 2 and Table S1). With regard to Cu, we know of soft tissue values for the European polecat (22.91 mg/kg), beech marten (7.87 mg/kg), the European badger (0.83 mg/kg), and red foxes (8.21 mg/kg) from Slovakia. However, it is unclear which tissue samples—from the aforementioned predator species—the values were detected from, and whether they refer to wet or dry weight [87].

No European results are known regarding the bioaccumulation of Mn, Se, and Cr in small mustelidae (Table S2). In Europe, Mn concentrations ranging from 3.01 to 4.72 mg/kg were documented in the livers of terrestrial predators with habitats and diet sources similar to those of the least weasel, while no data are available for kidneys (Table S2). The concentrations we found—regardless of tissue sample—are lower than the values published for terrestrial predators, with the greatest similarity to golden jackal samples from Croatia [76]. According to [88], the Mn content of tissues in mammals ranges from 0.3 to 2.9 mg/kg, which is consistent with our own results. Median values of 1.79–2.84 mg/kg were reported in the liver tissue of red foxes from an anthropogenically impacted area in the Czech Republic, which is similar to the concentration detected in the liver of the least weasel [89].

In Europe, Se concentration was determined primarily in liver tissue, where it ranges from 0.26 to 1.36 mg/kg. In our study, the average Se concentration measured in the liver also falls within this range. Data on Se content in kidneys are available for red foxes in Poland [90,91], which are lower than the kidney tissue concentration determined by us (Table 2 and Table S2). According to our test results, Se accumulates in greater quantities in the kidneys than in the liver, which was confirmed in several studies related to aquatic habitats [92,93,94,95], terrestrial [90,91], and domesticated predators [96]. The primary route of Se accumulation in tissues is through diet intake [97]. The transfer of Se through trophic levels was also documented [98]. Ref. [99] did not confirm the negative physiological effects of Se in his study, but due to its biomagnification properties, the effects of Se contamination must be interpreted holistically.

In predators that share similarities with the least weasel in terms of habitat and dietary biology, hepatic Cr accumulation was documented in the range of 0.02–0.38 mg/kg, which is lower than the amount accumulated in the least weasel. With regard to Cr content in the kidneys, concentrations several times lower were detected in red foxes (Table 2 and Table S2). In our study, we described a significant accumulation in kidney tissue, which is consistent with the tissue accumulation pattern observed in related species [94]. The higher values detected in the soft tissues of the least weasel can be explained by the fact that in mammals occupying higher trophic levels, the tissue concentration of Cr is equal to or lower than the accumulation values measured in taxa occupying lower trophic levels in the given habitat, contrary to the classic biomagnification pattern. Canines occupy a higher trophic level than the least weasel [79,80]. This phenomenon of biominification [100] was described in both terrestrial ecosystems [101] and aquatic ecosystems [102]. The explanation for this can be linked to the poor absorption of Cr in the digestive system [103].

We are not aware of any studies from Europe investigating Mo tissue concentrations in terrestrial mustelidae. Among predators inhabiting grasslands, the hepatic Mo concentrations detected in red foxes and golden jackals are lower than the values we measured in the same tissue (Table 2 and Table S3). We observed a more significant accumulation of Mo in liver samples compared to kidney samples, which was also confirmed in studies on other related species [92,94] and in a comprehensive study covering several mammalian species [104]. We found higher Mo concentrations in the least weasel than in related species, which can be explained by the location of the sample collection. The availability of Mo in the soil to plants is determined by the chemical properties of the soil [104,105]. The results of some studies suggest that Mo is mobile in trophic chains [106,107]. The vast majority of the least weasel individuals included in our study originate from the Kisalföld region of Hungary, where previous soil tests determined a pH value of 7.1–8.6 [108], which may result in more efficient availability and incorporation of Mo into the local biotic community.

We also have no data on Co accumulation in soft tissue in the least weasel. Significant Co accumulation was measured in red foxes in Hungary and Bulgaria, while in Bulgaria, concentrations several times higher than those identified by us were reported in golden jackals, and similar results were published in Croatia. The Co accumulation in liver tissue of European canine mesopredators ranged from 0.023 to 1.38 mg/kg, while the accumulation in kidney tissue ranged from 0.26 to 0.618 mg/kg on average. In the least weasel, we determined lower average values overall, regardless of tissue type (Table 2 and Table S3). In our study, we found significant accumulation in the kidneys, and similar and opposite tissue variability is also known in the literature (Table S3).

### 4.2. Accumulation Values of Non-Essential Elements: Comparison of Own Data with Data on Terrestrial Predators

In mammals, Cd, Pb, Hg, and Sb are considered non-essential elements [59,109,110,111], while there is no consensus on the essentiality of Ni, As, Sn, and V [59,112], and the essential role of Ti is also unknown [113].

There is little data available on Sn bioaccumulation values in carnivorous mammals. In red foxes from Poland, average values eight times higher in liver tissue and six times higher in kidney tissue were reported compared to the quantitative data measured in the same tissue in the least weasel. In our sample types, Sn was present in equal amounts in the analyzed samples, while in red foxes, stronger binding was detected in the liver (Table S4).

The reason for the low bioaccumulation of Ti may be that, compared to other heavy metals, it has poorer accumulation properties and is efficiently excreted from the body [114]. In our study, Ti was bound to the liver to a greater extent (Table 2).

Complex ecotoxicological studies were conducted on canines in several European countries, which also reported data on Ni accumulation. Significant concentrations were reported in red foxes from Hungary [82], Poland [84], Romania [85], and Bulgaria [86]. Similarly, in golden jackals, significantly higher concentrations were measured in Croatia [83], Romania [85], and Bulgaria [86]. In central Bulgaria, concentrations lower than and similar to our values were reported for golden jackals [115]. Based on these studies, Ni concentrations in liver tissue ranged from 0.007 to 3.74 mg/kg, while concentrations in kidney tissue ranged from 0.007 to 1.94 mg/kg. Our findings in the least weasel also fall within this range, and in fact, most studies reported significantly higher concentrations. In most studies, the nephritic Ni concentration is somewhat higher than the hepatic concentration (Table S4), which the authors attribute to dietary habits and agricultural practices [83,115]. Wild mammals can be contaminated with Ni through the digestive system, the integument, and the respiratory system, with food being the most important exposure factor [116]. According to [116], mammalian tissues contain this element at an average concentration of 0.1–4 mg/kg (dry weight), while in contaminated areas, Ni accumulation in tissues ranges from 0.5 to 10 mg/kg (dry weight). According to this study, Ni content is often higher in the kidneys than in liver tissue. This finding is confirmed by most of the studies we cited (Table S4), but we did not observe any quantitative variability between tissues (Table 2), and in some cases, higher accumulation was documented in liver tissue [117]. Following oral administration, it was observed that the absorption rate of soluble Ni compounds (NiSO_4_, NiCl_2_, and Ni(NO_3_)_2_) is much better than that of insoluble forms (NiO, Ni, Ni_3_S_2_, and NiS), and animal experiments documented that soluble compounds accumulate primarily in tissues responsible for detoxification and excretion (liver and kidneys), while insoluble forms accumulate in the lungs and pancreas [118].

Hg, along with Pb and Cd, is a toxic heavy metal that does not perform any physiological functions in mammals [119]. The toxicity of Hg is well known, which explains the widespread examination of the bioaccumulation of this heavy metal; accordingly, its presence in the soft tissues of terrestrial predators is well documented. In the European polecat from Switzerland and Finland, Hg concentrations were detected that were lower than those recorded in the least weasel from Poland. We also have data on this small predator from Slovakia (0.045 mg/kg; [87]). In central Italy, Hg concentrations in liver tissue of the beech marten were reported to be similar to those measured in our kidney samples, while in Croatia, lower concentrations were reported in individuals from both urban and rural habitats. In Croatia, lower Hg accumulation values were published for the European pine marten, while in Poland, the values were close to our results. We also have test results for the European pine marten from Slovakia (0.100 mg/kg; [87]). Similar or lower concentrations were measured in Italy, Poland, Croatia, and southern Spain [14] for the European badger. Values exceeding our results were reported from north-western Spain [23]. In addition, Hg accumulation in the soft tissues of the European badger was also studied in Slovakia (0.009 mg/kg; [87]). Among terrestrial mesopredators, lower concentrations than those identified in the tissues of the least weasel were published for the red fox from Italy, Spain, urban and rural environments in Croatia, and Poland. In Russia [120], however, Hg accumulation in the soft tissues of red foxes was more significant, and we also know of similar findings from Slovakia (0.147 mg/kg; [87]). In golden jackals from Croatia, Hg concentrations lower than our results were reported (Table S5). The Hg accumulation detected in liver samples from the aforementioned terrestrial predators ranged from 0.009 to 0.39 mg/kg, and 0.017–1.04 mg/kg in kidney tissue. In our study, the concentrations detected in the least weasel, regardless of the type of organ examined, fall within the extreme values determined in European predatory mammals (Table 2). Based on most of the studies cited, Hg accumulation is more significant in the kidneys than in the liver (Table S5), and we also identified this trend in the least weasel (Table 2). Terrestrial predators have lower tissue concentrations than semi-aquatic or aquatic species [17,94], which can be explained by the biomagnification properties of the element observed in aquatic ecosystems. This explains why higher concentrations were sometimes documented in the European polecat, which has a higher proportion of diet sources associated with aquatic habitats (Table S5) [121]. The lower Hg concentrations described in terrestrial predators can also be linked to the ban on Hg-containing fungicides [14].

Pb is the only toxic heavy metal known to accumulate in least weasel tissue, as [58] reported twice the concentration of Pb in liver tissue compared to the values obtained in our study. Among small mustelidae, higher Pb concentrations were reported in samples from the beech marten in central Italy and from urban and rural habitats in Croatia. In the European polecat, more significant Pb accumulation was documented in Switzerland and Italy than in our results, and accumulation values for this species are also known from Slovakia (0.284 mg/kg; [87]). Lower concentrations were detected in the European pine marten in Croatia and higher concentrations in Italy, but Pb tissue accumulation in this species was also studied in Slovakia (0.195 mg/kg; [87]). We have data from several countries on the concentration of Pb accumulating in the tissues of the European badger, which are larger member of the mustelidae family. Significant accumulation values were documented in the Netherlands, Italy, Spain, and Croatia, and we also have results for this species from Slovakia. (0.034 mg/kg; [87]). Among canines, higher Pb concentrations were detected in red foxes from Italy, Hungary, Poland, Romania, and Bulgaria, similar and lower concentrations were detected in Spain. Concentrations in this mesopredator are also known from a Slovakian study (1.137 mg/kg; [87]). In golden jackals, multiple Pb concentrations were determined in Serbia, Bulgaria, Croatia, and Romania. In the studies discussed, the average Pb concentration in the liver was determined to be in the range of 0.008–2.67 mg/kg, and the average Pb concentration in the kidneys was determined to be in the range of 0.009–2.12 mg/kg, with the vast majority of studies documenting higher Pb levels than our results. We found individual outliers in liver tissue that exceeded the upper limit of the aforementioned range (Table 2 and Table S5), which indicates the possibility of Pb exposure in the field habitat.

With regard to Cd bioaccumulation, higher average values than our own measurements were documented for the European polecat in Switzerland and Italy. Measurements were taken for this small predator in Slovakia (0.17 mg/kg; [87]). Significant concentrations were published for the beech marten in central Italy and in urban and rural areas of Croatia. Higher Cd concentrations were recorded in the European pine marten in Croatia and Italy, and data from Slovakia are also known (0.250 mg/kg; [87]). Several studies investigated the accumulation of Cd in the European badger, with exceptionally high values measured in kidney samples in the Netherlands, but significant Cd values are also known from Italy and Croatia. In comparison, lower concentrations were identified in samples from southern Spain [14] and higher concentrations in samples from the north-western part of the country [23] than in our own test results. Cd concentrations in the European badger were also studied in Slovakia (0.024 mg/kg; [87]). In red foxes, Cd concentrations exceeding our test results were identified in soft tissues in urban habitats in Croatia, Italy as well as in Hungary, Poland, Romania, and Bulgaria. In Spain and Croatia, similar levels of accumulation to those found in the least weasel in rural environments were published (Table S5), and test results were also reported from Slovakia (0.433 mg/kg; [87]) for this terrestrial predator. Based on studies of terrestrial carnivores, liver tissue concentrations in this taxon ranged from 0.0047 to 1.31 mg/kg, and kidney tissue accumulation ranged from 0.054 to 14.37 mg/kg. In our own study, we determined the average values for the least weasel soft tissue within this range, and most of the cited studies reported accumulation values exceeding our results (Table 2 and Table S5). Overall, it can be stated that this element accumulates in greater quantities in the kidney than in the liver, which we also confirmed in the least weasel.

With regard to V, we are not aware of any documented accumulation values in terrestrial mustelidae in tissue types corresponding to those in our study. Among canines, V accumulation was only measured in red foxes in Poland, where lower average concentrations were reported in liver and kidney tissue (0.055 mg/kg and 0.075 mg/kg) than those we detected in the same tissues of the least weasel. According to this study, the concentration of V was higher in the kidneys than in the liver (Table 2 and Table S6) [74]. We also confirmed this quantitative variability between tissues in our study.

The accumulation of Sb was also examined in a few studies. In small mammals from uncontaminated areas, the results are similar to ours, while significantly higher values were documented near Sb smelters [122]. In the case of Sb, we did not find any studies that examined the accumulation characteristics of the element in the soft tissues of small and medium-sized predator species in European countries. The low concentrations detected may be due to poor absorption through the intestinal tract, efficient excretion, low mobility in trophic chains, and low or no environmental exposure [122,123,124,125].

The bioaccumulation values of As were examined in Slovakia in the European polecat (0.236 mg/kg), the European pine marten (0.036 mg/kg), and the European badger (0.002 mg/kg) [87]. In Croatia, lower concentrations than our results were recorded in the European pine marten liver samples, while concentrations in kidney samples were similar. In Croatia, As concentrations in soft tissues of the beech marten from both urban and rural habitats were documented as being nearly identical to the concentrations we reported. In the case of the European badger, higher concentrations were identified in liver samples and similar concentrations in kidney samples in Croatia, while in Spain, significantly higher average concentrations were identified regardless of the organ. Several studies discussed the tissue As concentration in red foxes, reporting values in Spain and Croatia that are nearly identical to our results, regardless of tissue type and sample collection site. Higher accumulation values were published in Slovakia. However, low As concentrations were detected in golden jackal liver samples in Croatia. In carnivorous mammals, the concentration detected in the liver ranges from 0.0046 to 0.38 mg/kg, while in the kidney it ranges from 0.011 to 0.30 mg/kg (Table S6). In our study, the concentrations found in the same tissues of the least weasel are at the lower limit of the values recorded for terrestrial predators (Table 2). In small rodents, it was found that the As accumulation factor was below one, suggesting that As is less concentrated in small rodents. As a result, the negative health effects associated with As exposure may be more moderate in predators that feed on small mammals [126]. This may explain the lower As values in the small mammal-specialist least weasel compared to other predator species. In Hungary, low As concentrations were generally identified in the upper layers of the soil, which may also be a reason for the low values [127].

### 4.3. Anthropogenic Heavy Metal Pollution Affecting Agricultural Ecosystems

Due to the bioaccumulation and biomagnification properties of heavy metals, the entire biota of agricultural ecosystems may be affected by anthropogenic heavy metal pollution [128,129]. Since our samples originate from habitats affected by intensive agriculture, and we identified tissue accumulation that differed from the concentrations detected in related or identical habitats in predatory mammals in the case of certain elements, we considered it necessary to compile the agricultural practices that proved to be important inputs for the accumulation of the heavy metals we studied in agricultural ecosystems. Several studies have been published in recent years that draw attention to the importance of monitoring anthropogenic heavy metal loads in agricultural ecosystems [130,131]. In addition to agricultural chemicals such as pesticides and fertilizers used to increase productivity in these ecosystems, organic fertilizers, sewage, and the combustion of fossil fuels in agricultural machinery are the main sources of heavy metals. These sources and agricultural practices can also indirectly influence soil chemical parameters, thereby altering the availability of naturally occurring geogenic heavy metals in the affected habitats [42,132,133,134,135].

The use of fertilizers and organic manure in intensive agriculture is an important input in terms of heavy metal pollution in the area. Trace elements such as Fe, Zn, Cu, and Mn are added to fertilizers and introduced into the agricultural environment affected by intensive farming in order to eliminate trace element deficiencies in plants. Phosphate fertilizers have significant Cd, Co, Cu, and Zn content [33,132,136,137]. In addition, Cu is used as a food additive in pig farming, and the application of such organic fertilizers can also be an important route of exposure [138]. The positive effect of As-containing food additives on growth and their role in protecting against endoparasites have long been known, resulting in the use of As-containing additives in animal farming, which may contribute to the As load in the area through the application of animal-derived organic fertilizers to agricultural land [139]. In the field environment, anthropogenic Se loading is primarily associated with fertilization [140,141], similarly to Co, as the enrichment of this element, in addition to its geogenic origin, can be explained primarily by the use of organic and chemical fertilizers [142,143]. In addition to these elements, Mn [33,144], the six-valent (Hexavalent) form of Cr entered the environment almost entirely through anthropogenic means and is highly toxic; in mammals, Cr(VI) is reduced to Cr(III), forming toxic free radicals [145,146]. The Ni and Pb content of copper and iron sulfate fertilizers is well known [33]. The use of fertilizers also indirectly affects the availability of certain elements in agricultural areas. Fertilizers are responsible for a significant proportion of NH3 emissions [147], and ammonia was shown to contribute to the leaching of V from the soil, making it available to living organisms [133]. Fertilizers also contribute to changes in soil chemistry, affecting the availability of certain elements to biota [148]. This can be observed in the case of Mo, as Mo compounds dissolve poorly in acidic soils (<4.5) but well in alkaline soils (pH > 6.5), thus becoming available to plants in different ways [104,105]. According to [134], the levels of toxic elements such as Cd, As, and Pb increase in soils as a result of fertilizer use.

Another important source of anthropogenic heavy metal accumulation is the use of pesticides. Studies conducted in agricultural areas showed a positive correlation between long-term intensive plant protection and heavy metal pollution of the soil [149]. Various groups of pesticides—bactericides, herbicides, fungicides, insecticides, rodenticides—are known to contain heavy metal compounds [8,33,34,35,124,150,151,152]. Heavy metals applied with pesticides can enter plants not only from the soil but also through deposition on leaf surfaces [153] and can have negative physiological effects on vertebrate taxa at multiple levels of the food chain [154]. However, we are also aware of reports stating that critical toxic levels are not typical in crops grown on soils treated with fungicides [155]. The source of anthropogenic Hg pollution in agricultural environments is the use of pesticides, although their use has been declining in recent years due to their environmental toxicity [13,14,156]. Nevertheless, their residues may remain in the environment [157], and due to its good biomagnification properties, this element can enter the organisms of living beings [13]. Similarly, the use of certain As-containing pesticides was also restricted due to their environmental toxicity [158], but the effects of their accumulation may be noticeable for a long time. In line with EU regulations on chemical use, heavy metal pollution affecting the agricultural environment is becoming increasingly homogenized among member states, as the range of authorized active substances is essentially the same. For this reason, heavy metal pollution from other activities (e.g., industrial sources) may play a role in cases of more pronounced local differences. This also includes the issue of irrigation with wastewater. As an alternative irrigation method, it is important and appropriate in agriculture from an ecological and food safety perspective, but in addition to its positive effects, negative effects, such as changes in the physical and chemical parameters of the soil, were also documented [159]. This can also be linked to the heavy metal content of wastewater, as an increase in the concentration of essential and non-essential elements (e.g., Cr, Pb, Cu, Zn, Co, Mn, Mo, and Cd) in the topsoil can be observed as a result of long-term wastewater use [104,160,161,162].

The combustion of fossil fuels can also be considered a potential source of pollution, primarily responsible for increased concentrations of non-essential metals such as Cd and Pb. In rural areas, paved and unpaved roads can be important sources of contamination [32]. Although environmental levels of Pb are declining due to regulations on the use of Pb in fuel [138], lead bullets from hunting may be a specific route of exposure for predatory mammals [31]. Lead bullets containing Sb can potentially be a source of Sb contamination in areas affected by hunting [125].

Although our study on heavy metal accumulation in the least weasel fills a knowledge gap, we encountered several limitations, which, paradoxically, make our results even more valuable. It would have been desirable to compare the results by sex; however, the species’ capture characteristics did not allow this. It would also have been interesting to compare heavy metal burdens in samples from intensive and extensive agricultural environments, as well as urban areas. Unfortunately, this was not possible, as the species is protected in Hungary.

## 5. Conclusions

There are no previous studies on the presence of heavy metals in the least weasel populations, so our results are novel in the field of ecotoxicological research. Based on our findings, we believe that the least weasel can be an important biomonitoring species in agricultural habitats, as they are directly and indirectly exposed to anthropogenic pollution affecting agricultural ecosystems. Its specialist dietary strategy targeting small mammals also suggests that it may be an important terrestrial predator in ecotoxicological studies aimed at assessing heavy metal levels, as the small mammals they prey on absorb both essential and toxic heavy metals from their environment, and these elements can be a potential source of contamination for the predators that feed on them due to their biomagnification properties [163]. Terrestrial predators are much less studied than aquatic and semi-aquatic predators, and within this group, the least information is available on terrestrial small mustelidae. In our study, we also report quantitative data on the accumulation of several elements—Ti, V, Cr, Co, Ni, Se, Mo, Sb, and Sn—whose bioaccumulation values in mustelidae are understudied or completely lacking. With regard to Fe, bioaccumulation studies are primarily aimed at determining hepatic Fe content, so our nephritic values can be considered to fill a gap. Our results may serve as a guideline for terrestrial mustelidae after the normal and toxic threshold values for heavy metals have been determined. The intraspecific variability of heavy metals may depend not only on the geochemical background of the study sites, anthropogenic exposure, and duration of exposure, but also on physiological and ethological characteristics such as age, dietary habits, habitat selection, and sex. We consider it important to conduct regular biomonitoring of heavy metal concentrations in agricultural ecosystems for several species in order to obtain a more accurate picture of anthropogenic exposure pathways in the environment and the possible toxic mechanisms of action of the given element on populations. Our study on the least weasel fills a gap in this regard.

## Figures and Tables

**Table 1:** Measured isotopes, internal standards, limits of detection (LoD) of the elements, and quality control (QC) of the analytical measurements.

Element	Isotope (*m*/*z*)	Internal Standard Isotope	LoD (mg/kg)	QC (SRM 1577c) (n = 3)		
				Certified Value * (mg/kg)	Measured Value * (mg/kg)	Recovery ** (%)
Antimony (Sb)	121	In^115^	0.0065	0.00313 ± 0.00031	<0.0065	102 **
Arsenic (As)	75	Ge^74^	0.013	0.0196 ± 0.0014	0.0186 ± 0.0047	95
Cadmium (Cd)	111	In^115^	0.0037	0.0970 ± 0.0014	0.0958 ± 0.0050	99
Chromium (Cr)	52	Ge^74^	0.45	0.053 ± 0.014	<0.45	97 **
Cobalt (Co)	59	Ge^74^	0.0079	0.300 ± 0.018	0.280 ± 0.002	93
Copper (Cu)	65	Ge^74^	0.31	275.2 ± 4.6	259.0 ± 1.6	94
Iron (Fe)	56	Ge^74^	10	197.94 ± 0.65	180.1 ± 1.3	91
Lead (Pb)	207	Bi^209^	0.033	0.0628 ± 0.0010	0.0576 ±0.0006	92
Manganese (Mn)	55	Ge^74^	0.098	10.46 ± 0.47	9.58 ± 0.03	92
Mercury (Hg)	201	Bi^209^	0.025	0.00536 ± 0.00017	<0.025	112 **
Molybdenum (Mo)	95	In^115^	0.080	3.30 ± 0.13	3.25 ± 0.03	99
Nickel (Ni)	60	Ge^74^	0.089	0.0445 ± 0.0092	<0.089	98 **
Selenium (Se)	78	Ge^74^	0.024	2.031 ± 0.045	1.862 ± 0.027	92
Tin (Sn)	118	In^115^	0.043	N.A.	<0.043	92 **
Titanium (Ti)	48	Ge^74^	0.17	N.A.	0.293 ± 0.019	93 **
Vanadium (V)	51	Ge^74^	0.0079	0.00817 ± 0.00066	0.00877 ± 0.00012	107
Zinc (Zn)	68	Ge^74^	4.8	181.1 ± 1.0	183.1 ± 2.6	101

* Certified and measured values refer to dry mass concentrations. ** Spiked samples were used for those elements that did not have certified values in SRM 1577c, or the measured value in the CRM was below the LoD of the respective element. The spiked values were equal to 1.0 mg/kg. N.A. = not available.

**Table 2:** Standard descriptive statistics for Organ Type (liver and kidney) separately, irrespective of country. The values are expressed in mg/kg wet weight. Q1 designates the first quartile, while Q3 designates the third quartile, with Q1 representing the lower 25% of the observations when the data are arranged in ascending order. Q3 represents the upper quartile (also referred to as the third quartile or the 75th percentile of a dataset), containing 75% of the observations in the upper half of the data in an ordered dataset.

Organ Type	Variable	M	SD	Min	Q1	Median	Q3	Max
Liver	Ti	0.48	0.19	0.17	0.32	0.48	0.63	0.79
	V	0.01	0.02	0.01	0.01	0.01	0.01	0.18
	Cr	0.68	1.06	0.45	0.45	0.45	0.45	7.71
	Mn	2.68	0.88	1.22	2.0	2.55	3.26	4.77
	Fe	242.18	74.04	103.36	182.85	235.6	288.61	450.72
	Co	0.03	0.01	0.01	0.02	0.03	0.03	0.05
	Ni	0.11	0.15	0.09	0.09	0.09	0.09	1.31
	Cu	7.57	3.49	3.5	5.3	6.67	8.53	21.09
	Zn	43.31	21.18	18.97	30.35	36.46	43.85	139.34
	As	0.01	0.01	0.01	0.01	0.01	0.01	0.05
	Se	0.71	0.16	0.47	0.62	0.68	0.75	1.57
	Mo	0.89	0.2	0.44	0.78	0.87	1.01	1.37
	Sb	0.01	0.0	0.01	0.01	0.01	0.01	0.04
	Cd	0.02	0.03	0.0	0.01	0.01	0.02	0.15
	Sn	0.04	0.0	0.04	0.04	0.04	0.04	0.07
	Hg	0.07	0.23	0.02	0.02	0.02	0.02	1.83
	Pb	0.04	0.02	0.03	0.03	0.03	0.03	0.14
Kidney	Ti	0.18	0.04	0.17	0.17	0.17	0.17	0.47
	V	0.02	0.03	0.01	0.01	0.01	0.01	0.17
	Cr	0.73	1.24	0.45	0.45	0.45	0.45	7.86
	Mn	0.86	0.2	0.47	0.72	0.84	0.98	1.49
	Fe	144.15	36.28	67.72	119.24	140.3	167.29	263.9
	Co	0.04	0.01	0.02	0.03	0.03	0.04	0.06
	Ni	0.1	0.03	0.09	0.09	0.09	0.09	0.25
	Cu	7.89	2.84	2.98	5.84	7.27	9.91	19.27
	Zn	36.13	17.43	17.63	26.08	31.02	39.27	117.52
	As	0.01	0.0	0.01	0.01	0.01	0.01	0.03
	Se	1.11	0.28	0.67	0.93	1.06	1.21	2.43
	Mo	0.26	0.06	0.12	0.21	0.25	0.29	0.49
	Sb	0.01	0.01	0.01	0.01	0.01	0.01	0.08
	Cd	0.06	0.06	0.01	0.03	0.05	0.07	0.35
	Sn	0.04	0.0	0.04	0.04	0.04	0.04	0.04
	Hg	0.1	0.35	0.02	0.02	0.02	0.03	2.8
	Pb	0.06	0.13	0.03	0.03	0.03	0.03	1.04

Note. Number of paired specimens is 64.

**Table 3:** A comparison of liver vs. kidney metal concentration (mg/kg w.w.) in the sampled specimens. If the normal distribution holds, paired samples-t tests were conducted. Otherwise, the Wilcoxon signed-rank were performed. The Benjamini–Hochberg adjusted *p*-values (p_BH_adj) from these tests are reported. Liver data and kidney data are correlated samples, i.e., two values (one for the kidney and one for the liver) belong to the same animal.

Metal	M Liver	SD Liver	M Kidney	SD Kidney	p_BH_adj
Ti	0.47	0.18	0.18	0.04	*p* < 0.001
V	0.01	0.02	0.02	0.03	*p* = 0.44
Cr	0.68	1.07	0.74	1.25	*p* = 0.72
Mn	2.70	0.88	0.86	0.20	*p* < 0.001
Fe	243.48	73.89	144.44	36.49	*p* < 0.001
Co	0.02	0.01	0.04	0.01	*p* < 0.001
Ni	0.09	0.02	0.10	0.03	*p* = 0.44
Cu	7.61	3.50	7.85	2.85	*p* = 0.44
Zn	43.45	21.32	36.25	17.55	*p* < 0.001
As	0.01	0.01	0.01	0.00	*p* = 0.45
Se	0.71	0.16	1.11	0.28	*p* < 0.001
Mo	0.89	0.20	0.25	0.06	*p* < 0.001
Sb	0.01	0.00	0.01	0.01	*p* = 0.75
Cd	0.02	0.03	0.06	0.06	*p* < 0.001
Sn	0.04	0.00	0.04	0.00	*p* = 0.44
Hg	0.07	0.23	0.10	0.36	*p* < 0.001
Pb	0.04	0.02	0.06	0.13	*p* < 0.05

Note: “M” stands for sample mean, and “SD “for standard deviation of the sample. Significant differences in means are marked in bold. “p_BH_adj” designates Benjamini–Hochberg adjustment. Details about the use of parametric of non-parametric tests are not indicated in the table to save space. Positive values indicate higher hepatic accumulation, while negative values indicate higher renal accumulation.

**Table 4:** MCID (0.5 SD) Table for Significant Metals.

Metal	Mean_Diff	SD_Diff	MCID_0.5×SD	Exceeds_MCID
Ti	0.29	0.18	0.09	Yes
Mn	1.85	0.81	0.41	Yes
Fe	99.04	65.76	32.88	Yes
Co	−0.01	0.01	0.0	Yes
Zn	7.19	8.9	4.45	Yes
Se	−0.4	0.2	0.1	Yes
Mo	0.63	0.19	0.09	Yes
Cd	−0.04	0.04	0.02	Yes
Hg	−0.03	0.13	0.06	No
Pb	−0.02	0.12	0.06	No

Note. Equal sample sizes. “Mean_Diff” designates the mean difference between the average concentrations of metals in the liver and the kidney. “Within-subject” MCID is half the standard deviation of change within individuals (i.e., 0.5 × SD_difference_). Positive values indicate higher hepatic accumulation, while negative values indicate higher renal accumulation.

**Table 5:** Comparison of Austria vs. Hungary of the liver data. Between-subject comparisons (*t*-test or Mann–Whitney U) were performed. Parametric or non-parametric tests were used depending on the distribution of the data. Only the adjusted Benjamini–Hochberg *p*-values from these tests are reported (designated by “p_BH_adj”).

Metal	Mean Austria	SD Austria	Mean Hungary	SD Hungary	p_BH_adj	Significant
Ti	0.262	0.069	0.535	0.163	*p* < 0.05	Yes
V	0.028	0.05	0.01	0.005	0.80	No
Cr	1.249	2.134	0.515	0.328	0.66	No
Mn	2.601	0.903	2.708	0.886	0.88	No
Fe	251.076	76.733	239.687	73.867	0.72	No
Co	0.027	0.01	0.025	0.007	0.72	No
Ni	0.106	0.048	0.113	0.172	0.28	No
Cu	9.008	4.064	7.165	3.244	0.20	No
Zn	54.828	24.632	40.082	19.163	0.14	No
As	0.015	0.009	0.014	0.004	0.68	No
Se	0.772	0.245	0.69	0.128	0.56	No
Mo	0.823	0.205	0.905	0.191	0.56	No
Sb	0.007	0	0.007	0.004	0.90	No
Cd	0.028	0.041	0.021	0.021	0.77	No
Sn	0.043	0	0.044	0.004	0.77	No
Hg	0.171	0.481	0.036	0.04	0.75	No
Pb	0.034	0.004	0.039	0.019	0.77	No

Note. Austria had 14 specimens, while Hungary had 50.

**Table 6:** Comparison of Austria vs. Hungary of the kidney data. Shapiro–Wilk test results (W; Shapiro–Wilk statistic) and between-subject comparisons (*t*-test or Mann–Whitney U) are indicated.

Metal	Mean Austria	SD Austria	Mean Hungary	SD Hungary	BH Adjusted *p*	Significant
Ti	0.187	0	0.243	0.103	*p* > 0.99	No
V	0.036	0.064	0.026	0.039	*p* > 0.99	No
Cr	7.862	0	2.56	2.498	*p* > 0.99	No
Mn	0.905	0.238	0.845	0.182	0.9621	No
Fe	143.036	45.129	144.457	33.936	*p* > 0.99	No
Co	0.035	0.009	0.036	0.009	*p* > 0.99	No
Ni	0.247	0	0.178	0.051	*p* > 0.99	No
Cu	9.324	3.492	7.486	2.531	0.4728	No
Zn	46.073	18.851	33.352	16.136	0.4728	No
As	0.026	0	0.021	0.01	*p* > 0.99	No
Se	1.279	0.405	1.062	0.219	0.4728	No
Mo	0.28	0.072	0.249	0.06	0.637	No
Sb	0	0	0.038	0.034	*p* > 0.99	No
Cd	0.073	0.088	0.061	0.055	*p* > 0.99	No
Sn	0	0	0	0	*p* > 0.99	No
Hg	0.672	1.197	0.1	0.142	0.9621	No
Pb	0.055	0.026	0.17	0.277	0.637	No

Note. Austria had 14 specimens, while Hungary had 50.

## Data Availability

The original contributions presented in this study are included in the article/Supplementary Materials. Further inquiries can be directed to the corresponding author(s).

## Supplementary Materials

The following supporting information can be downloaded at: https://www.mdpi.com/article/10.3390/toxics14020118/s1, Table S1: Concentrations of Fe, Zn, and Cu in liver and kidney tissue in mustelidae and canine predators; Table S2: Concentrations of Mn, Se, and Cr in liver and kidney tissue in mustelidae and canine predators; Table S3: Concentration of Mo and Co in liver and kidney tissue in canine predators; Table S4: Concentrations of Sn, Ti, and Ni in liver and kidney tissue in canine predators; Table S5: Concentrations of Hg, Pb, and Cd in liver and kidney tissue in mustelidae and canine predators; Table S6: Concentrations of V, Sb, and As in liver and kidney tissue in mustelidae and canine predators. References [164,165,166,167] are cited in Supplementary Materials.

## References

[1] Duffus J.H. (2002). “Heavy metals” a meaningless term?. Pure Appl. Chem..

[2] Järup L. (2003). Hazards of heavy metal contamination. Br. Med. Bull..

[3] Verma R., Vijayalakshmy K., Chaudhiry V. (2018). Detrimental impacts of heavy metals on animal reproduction: A review. J. Entomol. Zool. Stud..

[4] Nriagu J.O. (1988). A Silent Epidemic of Environmental Metal Poisoning?. Environ. Pollut..

[5] Duruibe J.O., Ogwuegbu M.O.C., Egwurugwu J.N. (2007). Heavy metal pollution and human biotoxic effects. Int. J. Phys. Sci..

[6] Wuana R.A., Okieimen F.E. (2011). Heavy Metals in Contaminated Soils: A Review of Sources, Chemistry, Risks and Best Available Strategies for Remediation. ISRN Ecol..

[7] Gall J.E., Boyd R.S., Rajakaruna N. (2015). Transfer of heavy metals through terrestrial food webs: A review. Environ. Monit. Assess..

[8] Alengebawy A., Abdelkhalek S.T., Qureshi S.R., Wang M.-Q. (2021). Heavy Metals and Pesticides Toxicity in Agricultural Soil and Plants: Ecological Risks and Human Health Implications. Toxics.

[9] Govind P., Madhuri S. (2014). Heavy Metals Causing Toxicity in Animals and Fishes. Res. J. Anim. Vet. Fish. Sci..

[10] Tovar-Sánchez E., Hernández-Plata I., Martínez M.S., Valencia-Cuevas L., Galante P.M., Saleh H.M., Aglan R.F. (2018). Heavy Metal Pollution as a Biodiversity Threat. Heavy Metals.

[11] Mitra S., Chakraborty A.J., Tareq A.M., Emran T.B., Nainu F., Khusro A., Idris A.M., Khandaker M.U., Osman H., Alhumaydhi F.A. (2022). Impact of heavy metals on the environment and human health: Novel therapeutic insights to counter the toxicity. J. King Saud Univ. Sci..

[12] Garcia M.H.D., Moreno D.H., Rodriguez F.S., Beceiro A.L., Alvarez L.E.F., Lopez M.P. (2011). Sex- and age-dependent accumulation of heavy metals (Cd, Pb and Zn) in liver, kidney and muscle of roe deer (*Capreolus capreolus*) from NW Spain. J. Environ. Sci. Health A.

[13] Wren C.D. (1986). A Review of Metal Accumulation and Toxicity in Wild Mammals: I. Mercury. Environ. Res..

[14] Millán J., Mateo R., Taggart M.A., Lopez-Bao J.V., Viota M., Monsalve L., Camarero P.R., Blázquez E., Jiménez B. (2008). Levels of heavy metals and metalloids in critically endangered Iberian lynx and other wild carnivores from Southern Spain. Sci. Total Environ..

[15] Bilandžić N., Dežđek D., Sedak M., Dokić M., Solomun B., Varenina I., Knežević Z., Slavica A. (2010). Concentrations of Trace Elements in Tissues of Red Fox (*Vulpes vulpes*) and Stone Marten (*Martes foina*) from Suburban and Rural Areas in Croatia. Bull. Environ. Contam. Toxicol..

[16] Bilandžić N., Dežđek D., Sedak M., Dokić M., Simić B., Rudan N., Brstilo M., Lisicin T. (2012). Trace elements in tissues of wild carnivores and omnivores in Croatia. Bull. Environ. Contam. Toxicol..

[17] Lanszki J., Orosz E., Sugár L. (2009). Metal levels in tissues of Eurasian otters (*Lutra lutra*) from Hungary: Variation with sex, age, condition and location. Chemosphere.

[18] Kalisińska E., Lanocha-Arendarczyk N., Kosik-Bogacka D., Budis H., Podlasinska J., Popiolek M., Pirog A., Jedrzejewska E. (2016). Brains of Native and Alien Mesocarnivores in Biomonitoring of Toxic Metals in Europe. PLoS ONE.

[19] Goretti E., Pallottini M., Cenci Goga B.T., Selvaggi R., Petroselli C., Vercillo F., Cappelletti D. (2018). Mustelids as bioindicators of the environmental contamination by heavy metals. Ecol. Indic..

[20] Ljungvall K., Magnusson U., Korvela M., Norrby M., Bergquist J., Persson S. (2016). Heavy metal concentrations in female wild mink (*Neovison vison*) in Sweden: Sources of variation and associations with internal organ weights. Environ. Toxicol. Chem..

[21] Mason C.F., Weber D. (1990). Organochlorine Residues and Heavy Metals in Kidneys of Polecats (*Mustela putorius*) from Switzerland. Bull. Environ. Contam. Toxicol..

[22] Ozimec S., Florijancic T., Milin Radic S., Bilandzic N., Boskovic I. (2015). Bioaccumulation of cadmium and lead in the European badger (*Meles meles* L.) from the croatian danube region. J. Environ. Prot. Ecol..

[23] García-Muñoz J., Cacciola N.A., Plazzi F., Míguez-Santiyán M.P., Rodríguez F.S., López-Beceiro A., Fidalgo L.E., Martínez-Morcillo S., Pérez-López M. (2023). Metal and metalloid concentrations in wild mammals from SW Europe: European hedgehog (*Erinaceus europaeus*) and badger (*Meles meles*). Environ. Sci. Pollut. Res..

[24] Kim A., Woo D., Lee J.M., Kim J. (2023). Habitat use and preferences of the least weasel (*Mustela nivalis*) in South Korea. J. Ecol. Environ..

[25] Vass G., Bende A. (2024). The biology of the weasel (*Mustela nivalis* L.) in the light of the Hungarian literature. Magy. Apróvad Közlemények.

[26] Elmeros M. (2006). Food habits of stoats *Mustela erminea* and weasels *Mustela nivalis* in Denmark. Acta Theriol..

[27] Sidorovich V.E., Polozov A.G., Solovej I.A. (2008). Niche separation between the weasel *Mustela nivalis* and the stoat *M. erminea* in Belarus. Wildl. Biol..

[28] Vass G., Fekete I., Bende A. (2025). Nutritional biology of the weasel (*Mustela nivalis* L., 1766) in the light of the literature. North-West. J. Zool..

[29] Korpimäki E., Norrdahl K., Rinta-Jaskari T. (1991). Responses of stoats and least weasels to fluctuating food abundances: Is the low phase of the vole cycle due to mustelid predation?. Oecologia.

[30] Goszczyński J. (1999). Food composition of weasels (*Mustela nivalis*) in Poland. Mammalia.

[31] Pérez-López M., Rodríguez F.S., Hernández-Moreno D., Rigueira L., Fidalgo L.E., Beceiro A.L. (2015). Bioaccumulation of cadmium, lead and zinc in liver and kidney of red fox (*Vulpes vulpes*) from NW Spain: Influence of gender and age. Toxicol. Environ. Chem..

[32] Karaoğlu M., Küçük C. (2021). The Determination of Lead and Cadmium Concentration in the Agricultural Soils Alongside Highway 080 of Igdir Province. J. Agric..

[33] Gimeno-García E., Andreau V., Boluda R. (1996). Heavy metals incidence in the application of inorganic fertilizers and pesticides to rice farming soils. Environ. Pollut..

[34] Alnuwaiser M.A. (2019). An Analytical Survey of Trace Heavy Elements in Insecticides. Int. J. Anal. Chem..

[35] Kalayci Ş. (2022). Investigation of Arsenic Content in Field Pesticides Using Inductively Coupled Plasma Optical Emission Spectroscopy (ICP-OES). Gazi Univ. J. Sci. Part A Eng. Innov..

[36] Kalisińska E., Budis H., Kalisińska E. (2019). Manganese, Mn. Mammals and Birds as Bioindicators of Trace Element Contaminations in Terrestrial Environments.

[37] Oroszi S. (1996). Vadfajokból védett állatfajok. Erd. Köz..

[38] Jagd-Burgenland. https://www.jagd-burgenland.at/jagd/info/schusszeiten.

[39] Andesrecht Konsolidiert Burgenland Gesamte Rechtsvorschrift für Bgld. Jagdgesetz 2004, Fassung vom 29 March 2016. https://www.ris.bka.gv.at/.

[40] King C.M. (1975). The Home Range of the Weasel (*Mustela nivalis*) in an English Woodland. J. Anim. Ecol..

[41] Brandt M.J., Lambin X. (2007). Movement patterns of a specialist predator, the weasel *Mustela nivalis* exploiting asynchronous cyclic field vole *Microtus agrestis* populations. Acta Theriol..

[42] Rashid A., Schutte B.J., Ulery A., Deyholos M.K., Sanogo S., Lehnhoff E.A., Beck L. (2023). Heavy Metal Contamination in Agricultural Soil: Environmental Pollutants Affecting Crop Health. Agronomy.

[43] Keszthelyi G., Vass G., Faragó S., Bende A. (2024). Trapping results for Weasel (*Mustela nivalis*) and Norway rat (*Rattus norvegicus*) in the LAJTA Project. Magy. Apróvad Közlemények.

[44] King C.M. (1980). Population biology of the weasel *Mustela nivalis* on British game estates. Holarct. Ecol..

[45] McDonald R.A., Harris S. (2002). Population biology of stoats *Mustela erminea* and weasels *Mustela nivalis* on game estates in Great Britain. J. Appl. Ecol..

[46] R Core Team (2023). R: A Language and Environment for Statistical Computing (Version 4.3.1) [Computer Software]. R Foundation for Statistical Computing. https://www.r-project.org/.

[47] Hair J.F., Black W.C., Babin B.J., Anderson R.E. (2019). Multivariate Data Analysis.

[48] Benjamini Y., Hochberg Y. (1995). Controlling the false discovery rate: A practical and powerful approach to multiple testing. J. R. Stat. Soc. Ser. B Methodol..

[49] Mouelhi Y., Jouve E., Castelli C., Gentile S. (2020). How is the minimal clinically important difference established in health-related quality of life instruments? Review of anchors and methods. Health Qual. Life Outcomes.

[50] Norman G.R., Sloan J.A., Wyrwich K.W. (2003). Interpretation of changes in health-related quality of life: The remarkable universality of half a standard deviation. Med. Care.

[51] Fekete I. (2025). Transforming Data into Informed Decisions Across Clinical and Non-Clinical Domains.

[52] Sloan J.A., Cella D., Hays R.D. (2005). Clinical significance of patient-reported questionnaire data: Another step toward consensus. J. Clin. Epidemiol..

[53] Copay A.G., Subach B.R., Glassman S.D., Polly D.W., Schuler T.C. (2007). Understanding the minimum clinically important difference: A review of concepts and methods. Spine J..

[54] Wyrwich K.W., Nienaber N.A., Tierney W.M., Wolinsky F.D. (1999). Linking clinical relevance and statistical significance in evaluating intra-individual changes in health-related quality of life. Med. Care.

[55] Revicki D., Hays R.D., Cella D., Sloan J. (2008). Recommended methods for determining responsiveness and minimally important differences for patient-reported outcomes. J. Clin. Epidemiol..

[56] Kim J., Park J.H., Kim T.K. (2025). Analysis of interaction effect between within- and between-subject factors in repeated measures analysis of variance for longitudinal data. Korean J. Anesthesiol..

[57] Rencher A.C. (2002). Methods of Multivariate Analysis.

[58] Alleva E., Francia N., Pandolfi M., De Marinis A.M., Chiarotti F., Santucci D. (2006). Organochlorine and Heavy-Metal Contaminants in Wild Mammals and Birds of Urbino-Pesaro Province, Italy: An Analytic Overview for Potential Bioindicators. Arch. Environ. Contam. Toxicol..

[59] Kalisińska E. (2019). Mammals and Birds as Bioindicators of Trace Element Contaminations in Terrestrial Environments: An Ecotoxicological Assessment of the Northern Hemisphere.

[60] Goldyn B., Hromada M., Surmacki A., Tryjanowski P. (2003). Habitat use and diet of the red fox *Vulpes vulpes* in an agricultural landscape in Poland. Z. Jagdwiss..

[61] Červinka J., Šálek M., Padyšáková E., Šmilauer P. (2013). The effects of local and landscape-scale habitat characteristics and prey availability on corridor use by carnivores: A comparison of two contrasting farmlands. J. Nat. Conserv..

[62] Šálek M., Červinka J., Banea O.C., Krofel M., Ćirović D., Selanec I., Penezić A., Grill S., Riegert J. (2014). Population densities and habitat use of the golden jackal (*Canis aureus*) in farmlands across the Balkan Peninsula. Eur. J. Wildl. Res..

[63] Baghli A., Walzberg C., Verhagen R. (2005). Habitat use by the European polecat *Mustela putorius* at low density in a fragmented landscape. Wildl. Biol..

[64] Malecha A.W., Antczak M. (2013). Diet of the European polecat *Mustela putorius* in an agricultural area in Poland. Folia Zool..

[65] Romanowski J., Lesiński G. (1991). A note on the diet of stone marten in southeastern Romania. Acta Theriol..

[66] Zalewski A., Harrison D.J., Fuller A.K., Proulx G. Geographical and Seasonal Variation in Food Habits and Prey Size of European Pine Martens. Martens and Fishers (Martes) in Human-Altered Environments.

[67] Balestrieri A., Remonti L., Ruiz-González A., Vergara M., Capelli E., Gómez-Moliner B.J., Prigioni C. (2011). Food habits of genetically identified pine marten *(Martes martes)* expanding in agricultural lowlands (NW Italy). Acta Theriol..

[68] Goszczyński J., Jedrzejewska B., Jedrzejewski W. (2000). Diet composition of badgers (*Meles meles*) in a pristine forest and rural habitats of Poland compared to other European populations. J. Zool..

[69] Lanszki J., Kurys A., Szabó L., Nagyapáti N., Porter L.B., Heltai M. (2016). Diet composition of the golden jackal and the sympatric red fox in an agricultural area (Hungary). Folia Zool..

[70] Markov G., Lanszki J. (2012). Diet composition of the golden jackal, Canis aureus in an agricultural environment. Folia Zool..

[71] Lange P.N.A.M.J.G., Lelieveld G., De Knegt H.J. (2021). Diet Composition of the Golden Jackal (*Canis aureus*) in South-East Europe—A Review. Mammal Rev..

[72] Jahan Rakib M.R., Jolly Y.N., Enyoh C.E., Khandaker M.U., Hossain M.B., Akther S., Alsubaie A., Almalki A.S.A., Bradley D.A. (2021). Levels and health risk assessment of heavy metals in dried fish consumed in Bangladesh. Sci. Rep..

[73] Kalisińska E., Lisowski P., Salicki W., Kucharska T., Kavetska K. (2009). Mercury in wild terrestrial carnivorous mammals from north-western Poland and unusual fish diet of red fox. Acta Theriol..

[74] Kalisińska E., Kot K., Łanocha-Arendarczyk N. (2023). Red fox as a potential bioindicator of metal contamination in a European environment. Chemosphere.

[75] Lobanov A.V., Hatfield D.L., Gladyshev V.N. (2008). Reduced reliance on the trace element selenium during evolution of mammals. Genome Biol..

[76] Lazarus M., Sekovanić A., Orct T., Reljić S., Kusak J., Jurasović J., Huber Đ. (2017). Apex predatory mammals as bioindicator species in environmental monitoring of elements in Dinaric Alps (Croatia). Environ. Sci. Pollut. Res. Int..

[77] González-Montaña J.R., Escalera-Valente F., Alonso A.J., Lomillos J.M., Robles R., Alonso M.E. (2020). Relationship between Vitamin B12 and Cobalt Metabolism in Domestic Ruminant: An Update. Animals.

[78] Kosik-Bogacka D., Łanocha-Arendarczyk N., Kalisińska E., Kot K., Czernomysy-Furowicz D., Pilarczyk B., Tomza-Marciniak A., Kalisińska E. (2019). Iron, Fe. Mammals and Birds as Bioindicators of Trace Element Contaminations in Terrestrial Environments.

[79] Jędrzejewski W., Jędrzejewska B. (1992). Foraging and diet of the red fox *Vulpes vulpes* in relation to variable food resources in Biatowieza National Park, Poland. Ecography.

[80] Bakaloudis D., Bontzorlos V., Vlachos C., Papakosta M., Chatzinikos E., Braziotis S., Kontsiotis V. (2015). Factors affecting the diet of the red fox (*Vulpes vulpes*) in a heterogeneous Mediterranean landscape. Turk. J. Zool..

[81] Hatlauf J., Lanszki J. (2024). First dietary assessment of a generalist mesocarnivore, the golden jackal (*Canis aureus*) in Austria. Mamm. Biol..

[82] Heltai M., Markov G. (2012). Red Fox (*Vulpes vulpes* Linnaeus, 1758) as Biological Indicator for Environmental Pollution in Hungary. Bull. Environ. Contam. Toxicol..

[83] Ćirović D., Gizejewska A., Jovanović V., Penezić A., Milenković M., Vujošević M., Blagojević J. (2015). Concentration of selected trace elements in the golden jackal (*Canis aureus* L.,1758) population from Serbia. Acta Zool. Bulg..

[84] Binkowski Ł.J., Merta D., Przystupińska A., Sołtysiak Z., Pacon J., Stawarz R. (2016). Levels of metals in kidney, liver and muscle tissue and their relation to the occurrence of parasites in the red fox in the Lower Silesian forest in Europe. Chemosphere.

[85] Farkas A., Bidló A., Bolodár-Varga B., Jánoska F. (2017). Accumulation of metals in liver tissues of sympatric golden jackal (*Canis aureus*) and red fox (*Vulpes vulpes*) in the southern part of Romania. Bull. Environ. Contam. Toxicol..

[86] Georgiev D., Raichev E., Dospatliev L., Ivanova M., Peeva S., Kalcheva S., Georgieva K. (2018). Heavy metals concentrations in organs of red foxes (*Vulpes vulpes* Linnaeus, 1758) and golden jackals (*Canis aureus* Linnaeus, 1758) inhabiting the “Sarnena Sredna Gora” mountain in Bulgaria. Bulg. J. Agric. Sci..

[87] Maľová J., Ciberej J., Maľa P., Zigo F., Semjon B. (2019). Heavy metal levels in the tissues of wild living animals from two distinct industrially exploited areas in Slovakia. Slovak J. Anim. Sci..

[88] Aschner M., Erikson K. (2017). Manganese. Adv. Nutr..

[89] Jankovská I., Miholová D., Bejcek V., Vadlejch J., Sulc M., Száková J., Langrová I. (2010). Influence of parasitism on trace element contents in tissues of Red Fox (*Vulpes vulpes*) and its parasites *Mesocestoides* spp. (Cestoda) and *Toxascaris leonina* (Nematoda). Arch. Environ. Contam. Toxicol..

[90] Balicka-Ramisz A., Pilarczyk B., Ramisz A., Pilarczyk R., Nader K. (2010). Selenium concentrations in the liver, kidneys, and muscles in silver foxes [*Vulpes vulpes*]. Bull. Vet. Inst. Pulawy.

[91] Pilarczyk B., Pilarczyk R., Tomza-Marciniak A., Hendzel D., Bąkowska M., Stankiewicz T. (2011). Evaluation of selenium status and its distribution in organs of free living foxes (*Vulpes vulpes*) from an Se deficient area. Pol. J. Vet. Sci..

[92] Harding L.E., Harris M.L., Elliott J.E. (1998). Heavy and trace metals in wild mink (*Mustela vison*) and river otter (*Lontra canadensis*) captured on rivers receiving metals discharges. Bull. Environ. Contam. Toxicol..

[93] Gamberg M., Boila G., Stern G., Roach P. (2005). Cadmium, mercury and selenium concentrations in mink (*Mustela vison*) from Yukon, Canada. Sci. Total Environ..

[94] Brzeziński M., Zalewski A., Niemczynowicz A., Jarzyna I., Suska-Malawska M. (2014). The use of chemical markers for the identification of farm escapees in feral mink populations. Ecotoxicology.

[95] Kang S., Kang J.H., Kim S., Lee S.H., Lee S., Yu H.J., Oh S.J., Park J.D., Nam K.H., Han S.Y. (2015). Trace element analysis of three tissues from Eurasian otters (*Lutra lutra*) in South Korea. Ecotoxicology.

[96] Kot K., Piekara J., Kosik-Bogacka D., Łanocha-Arendarczyk N., Budis H., Pilarczyk B., Tomza-Marciniak A., Ciosek Z., Kalisińska E. (2017). Selenium levels in organs and tissues of domestic dog (*Canis lupus familiaris*) from the northwest area of Poland. Pomeranian J. Life Sci..

[97] Navarro-Alarcon M., Cabrera-Vique C. (2008). Selenium in food and the human body: A review. Sci. Total Environ..

[98] Hopkins A.H., Staub B.P., Baionno J.A., Jackson B.P., Talent L.G. (2005). Transfer of selenium from prey to predators in a simulated terrestrial food chain. Environ. Pollut..

[99] Clark D.R. (1987). Selenium accumulation in mammals exposed to contaminated California irrigation drainwater. Sci. Total Environ..

[100] Setmire J.G., Schroeder R.A., Densmore J.N., Goodbred S.L., Audet D.J., Radke W.R. (1993). Detailed Study of Water Quality, Bottom Sediment, and Biota Associated with Irrigation Drainage in the Salton Sea Area, California, 1988–90.

[101] Taylor F.G., Parr P.D., Dahlmann R.C. (1978). Distribution of chromium in vegetation and small mammals adjacent to cooling towers. J. Tenn. Acad. Sci..

[102] Morris R.J., Law R.J., Allchin C.R., Kelly C.A., Fileman C.F. (1989). Metals and Organochlorines in Dolphins and Porpoises of Cardigan Bay, West Wales. Mar. Pollut. Bull..

[103] Outridge P.M., Scheuhammer A.M. (1993). Bioaccumulation and Toxicology of Chromium: Implications for Wildlife. Rev. Environ. Contam. Toxicol..

[104] Kośla T., Skibniewski M., Skibniewska E.M., Lasocka I., Kołnierzak M., Kalisińska E. (2019). Molybdenum, Mo. Mammals and Birds as Bioindicators of Trace Element Contaminations in Terrestrial Environments.

[105] Rutkowska B., Szulc W., Spychaj-Fabisiak E., Pior N. (2017). Prediction of molybdenum availability to plants in differentiated soil conditions. Plant Soil Environ..

[106] Anke M., Seifert M., Holzinger S., Müller R., Schäfer U. (2007). The biological and toxicological importance of molybdenum in the environment and in the nutrition of plants, animals and man. Part 2: Molybdenum in animals and man. Acta Biol. Hung..

[107] Solaiman S.G., Beguesse K.A., Min B.R. (2024). Effect of High Molybdenum Diet on Copper Status, Growth Performance, Blood Metabolites, Select Liver and Kidney Minerals, and Immune Responses of Boer Crosses. Animals.

[108] László R., Heil B., Faragó S. (2012). A Lajta Project talajviszonyai. Lajta Project. Egy Tartamos Mezei Vad és Ökoszisztéma Vizsgálat 20 Éve.

[109] Filella M., Belzile N., Lett M.-C. (2007). Antimony in the environment: A review focused on natural waters. III. Microbiota relevant interactions. Earth-Sci. Rev..

[110] Vidya C.S.-N., Shetty R., Vaculíkova M., Vaculík M. (2022). Antimony toxicity in soils and plants, and mechanisms of its alleviation. Environ. Exp. Bot..

[111] Lazarus M., Sekovanić A., Reljić S., Kusak J., Ferenčaković M., Sindičić M., Gomerčić T., Huber Đ. (2023). Lead and Other Trace Element Levels in Brains of Croatian Large Terrestrial Carnivores: Influence of Biological and Ecological Factors. Toxics.

[112] Szentmihályi K., Klébert S., Somogyi A. (2022). Diabetes és a nyomelemek. Orv. Hetil..

[113] Zierden M.R., Valentine A.M. (2016). Contemplating a role for titanium in organisms. Metallomics.

[114] Farrel T.P., Magnuson B. (2017). Absorption, Distribution and Excretion of Four Forms of Titanium Dioxide Pigment in the Rat. J. Food Sci..

[115] Markov G., Kocheva M., Gospodinova M. (2016). Assessment of Heavy Metal Accumulation in the Golden Jackal (*Canis aureus*) as a Possible Bioindicator in an Agricultural Environment in Bulgaria. Bull. Environ. Contam. Toxicol..

[116] Outridge P.M., Scheuhammer A.M. (1993). Bioaccumulation and toxicology of nickel: Implications for wild mammals and birds. Environ. Rev..

[117] Severa J., Vyskocil A., Fiala Z., Cizkova M. (1995). Distribution of nickel in body fluids and organs of rats chronically exposed to nickel sulphate. Hum. Exp. Toxicol..

[118] Casalegno C., Schifanella O., Zennaro E., Marroncelli S., Chemservice S. (2015). Collate literature data on toxicity of chromium (Cr) and nickel (Ni) in experimental animals and humans. EFSA Support. Publ..

[119] Kalisińska E., Łanocha-Arendarczyk N., Kosik-Bogacka D.I., Kalisińska E. (2019). Mercury, Hg. Mammals and Birds as Bioindicators of Trace Element Contaminations in Terrestrial Environments.

[120] Piskorová L., Vasilková Z., Krupicer I. (2003). Heavy metal residues in tissues of wild boar (*Sus scrofa*) and red fox (*Vulpes vulpes*) in the Central Zemplin region of the Slovak Republic. Czech J. Anim. Sci..

[121] Hoekstra P.F., Braune B.M., Elkin B., Armstrong F.A., Muir D.C. (2003). Concentrations of selected essential and non-essential elements in arctic fox (*Alopex lagopus*) and wolverines (*Gulo gulo*) from the Canadian Arctic. Sci. Total Environ..

[122] Ainsworth N., Cokke J.A., Johnson M.S. (1990). Distribution of Antimony in Contaminated Grassland: 2-Small Mammals and Invertebrates. Environ. Pollut..

[123] Felicetti S.A., Thomas R.G., McClellan R.O. (1974). Metabolism of Two Valence States of Inhaled Antimony in Hamsters. Am. Ind. Hyg. Assoc. J..

[124] Puls R. (1994). Mineral Levels in Animal Health. Diagnostic Data.

[125] Bredsdorff L., Nielsen E. (2015). Antimony Evaluation of Health Hazards and Proposal of a Health Based Quality Criterion for Soil.

[126] Erry B.V., Macnair M.R., Meharg A.A., Shore R.F. (2000). Arsenic contamination in wood mice (*Apodemus sylvaticus*) and bank voles (*Clethrionomys glareolus*) on abandoned mine sites in southwest Britain. Environ. Pollut..

[127] Tóth G., Hermann T., Da Silva M.R., Montanarella L. (2016). Heavy metals in agricultural soils of the European Union with implications for food safety. Environ. Int..

[128] Zhang H., Zhao Y., Wang Z., Liu Y. (2021). Distribution characteristics, bioaccumulation and trophic transfer of heavy metals in the food web of grassland ecosystems. Chemosphere.

[129] Cai J., Zeng Y., Zhu Y., Zheng Q., Tian L., Xie O., Zheng X. (2024). Trophic stoichiometry of macroelements and metals in a terrestrial food web. Environ. Pollut..

[130] Oruc H.H., Cengiz M., Beskaya A. (2009). Chronic Copper Toxicosis in Sheep Following the Use of Copper Sulfate as a Fungicide on Fruit Trees. J. Vet. Diagn. Investig..

[131] Ellwanger J.H., Ziliotto M., Chies J.A.B. (2025). Impacts of Metals on Infectious Diseases in Wildlife and Zoonotic Spillover. J. Xenobiot..

[132] He Z.L., Yang X.E., Stoffella P.J. (2005). Trace elements in agroecosystems and impacts on the environment. J. Trace Elem. Med. Biol..

[133] Mandiwana K.L., Panichev N. (2009). The leaching of vanadium(V) in soil due to the presence of atmospheric carbon dioxide and ammonia. J. Hazard. Mater..

[134] Atafar Z., Mesdaghinia A., Nouri J., Homaee M., Yunesian M., Ahmadimoghaddam M., Mahvi A.H. (2010). Effect of fertilizer application on soil heavy metal concentration. Environ. Monit. Assess..

[135] Wan Y., Liu J., Zhuang Z., Wang Q., Li H. (2024). Heavy Metals in Agricultural Soils: Sources, Influencing Factors, and Remediation Strategies. Toxics.

[136] Lambert R., Grant C., Sauvé S. (2007). Cadmium and zinc in soil solution extracts following the application of phosphate fertilizers. Sci. Total Environ..

[137] Tomza-Marciniak A., Pilarczyk B., Marciniak A., Udała J., Bąkowska M., Pilarczyk R., Kalisińska E. (2019). Cadmium, Cd. Mammals and Birds as Bioindicators of Trace Element Contaminations in Terrestrial Environments.

[138] Van den Brink N.W., Ma W.-C. (1998). Spatial and temporal trends in levels of trace metals and PCBs in the European badger *Meles meles* L., 1758 in The Netherlands: Implications for reproduction. Sci. Total Environ..

[139] Garbarino J.R., Bednar A.J., Rutherford D.W., Beyer R.S., Wershaw R.L. (2003). Environmental Fate of Roxarsone in Poultry Litter. I. Degradation of Roxarsone during Composting. Environ. Sci. Technol..

[140] Korkman J. (1980). The effect of selenium fertilizers on the selenium content of barley, spring wheat and potatoes. Agric. Food Sci..

[141] De Feudis M., D’Amato R., Businelli D., Guiducci M. (2018). Fate of selenium in soil: A case study in a maize (*Zea mays* L.) field under two irrigation regimes and fertilized with sodium selenite. Sci. Total Environ..

[142] Shahbazi A., Soffianian A.R., Mirghaffari N., Rezaei H. (2018). Impact of agricultural activities on accumulation of Cadmium, Cobalt, Chromium, Copper, Nickel and Lead in soil of Hamedan province. Environ. Res. Res..

[143] Pan Y., Li X., Chen M., Wang X., Leng Y. (2025). Driving factors and spatial patterns of cobalt in agricultural soils of a karst area under the combined influence of geology and agricultural activities: A case study of Zhijin County, China. J. Hazard. Mater..

[144] Wyszkowski M., Brodowska M.S. (2021). Potassium and Nitrogen Fertilization vs. Trace Element Content of Maize (*Zea mays* L.). Agriculture.

[145] Ertani A., Mietto A., Borin M., Nardi S. (2017). Chromium in Agricultural Soils and Crops: A Review. Water Air Soil Pollut..

[146] Kośla T., Lasocka I., Kołnierzak M., Kalisińska E. (2019). Chromium, Cr. Mammals and Birds as Bioindicators of Trace Element Contaminations in Terrestrial Environments.

[147] Bouwman A.F., Van Der Hoek K.W. (1997). Scenarios of animal waste production and fertilizer use and associated ammonia emission for the developing countries. Atmos. Environ..

[148] Zhang L., Zhao Z., Jiang B., Baoyin B., Cui Z., Wang H., Li Q., Cui J. (2024). Effects of Long-Term Application of Nitrogen Fertilizer on Soil Acidification and Biological Properties in China: A Meta-Analysis. Microorganisms.

[149] Da Silva Martins T., Garcia K.G.V., da Silva Y.J.A.B., da Silva M.G., Serpa S.S.E., Bezerra R.A., Filho C.D.T., Cavalcante R.M., Boechat C.L., de Araujo Pereira A.P. (2024). Contamination risk by heavy metals and enzymatic stoichiometry in agricultural soils under intense use of pesticides. Environ. Monit. Assess..

[150] Kimbrough R.D. (1976). Toxicity and Health Effects of Selected Organotin Compounds: A Review. Environ. Health Perspect..

[151] Doyle J.J., Spaulding J.E. (1978). Toxic and essential trace elements in meat—A review. J. Anim. Sci..

[152] Akhtar M., Trombetta L.D. (2023). Low level mancozeb exposure causes copper bioaccumulation in the renal cortex of rats leading to tubular injury. Environ. Toxicol. Pharmacol..

[153] Mattielo A., Novello N., Cornu J.-Y., Babst-Kostecka A., Pošćić F. (2023). Copper accumulation in five weed species commonly found in the understory vegetation of Mediterranean vineyards. Environ. Pollut..

[154] Habuštová O., Weismann L., Harangozó M., Bumbálová A. (2000). Influence of copper from the Kuprikol 50 fungicide on Colorado potato beetle adults studied by radionuclide X-ray fluorescence analysis. J. Radioanal. Nucl. Chem..

[155] Ruyters S., Salaets P., Oorts K., Smolders E. (2012). Copper toxicity in soils under established vineyards in Europe: A survey. Sci. Total Environ..

[156] Hylander L.D., Meili M. (2003). 500 years of mercury production: Global annual inventory by region until 2000 and associated emissions. Sci. Total Environ..

[157] Lodenius M., Skaren U., Hellstedt P., Tulisalo E. (2014). Mercury in various tissues of three mustelid and other trace metals in liver o European otter from Eastern Finland. Environ. Monit. Assess..

[158] Gupta P.K., Gupta R.C., Gupta R.C. (2025). Toxicity of Herbicides. Veterinary Toxicology. Basic and Clinical Principles.

[159] Jaramillo M.F., Restrepo I. (2017). Wastewater Reuse in Agriculture: A Review about Its Limitations and Benefits. Sustainability.

[160] Murtaza G., Ghafoor A., Qadir M. (2008). Accumulation and implications of cadmium, cobalt and manganese in soils and vegetables irrigated with city effluent. J. Sci. Food Agric..

[161] Zojaji F., Hassani A.H., Sayadi M.H. (2014). Bioaccumulation of chromium by *Zea mays* in wastewater-irrigated soil: An experimental study. Proc. Int. Acad. Ecol. Environ. Sci..

[162] Alvarez-Holguin A., Sosa-Perez G., Ponce-Garcia O.C., Lara-Macias C.R., Villarreal-Guerrero F., Monzon-Burgos C.G., Ochoa-Rivero J.M. (2022). The Impact of Treated Wastewater Irrigation on the Metabolism of Barley Grown in Arid and Semi-Arid Regions. Int. J. Environ. Res. Public Health.

[163] Sánchez-Chardi A., Peñarroja-Matutano C., Ribeiro C.A.O., Nadal N. (2007). Bioaccumulation of metals and effects of a landfill in small mammals. Part II. The wood mouse, *Apodemus sylvaticus*. Chemosphere.

[164] Diethart N., Deutz A., Bauer S., Paulsen P. (2024). Chemical composition and selected element contents of livers from wild game hunted in Austria. J. Food Saf. Food Qual..

[165] Farkas A., Bidló A., Bolodár-Varga B., Jánoska F. (2021). Accumulation of selected metals and concentration of macroelements in liver and kidney tissues of sympatric golden jackal (*Canis aureus*) and red fox (*Vulpes vulpes*) in Somogy County, Hungary. Environ. Sci. Pollut. Res. Int..

[166] Corsolini S., Focardi S., Leonzio C., Lovari S., Monaci F., Romeo G. (1997). Heavy metals and chlorinated hydrocarbon concentrations in the red fox in relation to some biological parameters. Environ. Monit. Assess..

[167] Khabarova L.S., Poddubnaya N.Y., Selezneva A.P., Ivanova E.S., Andreeva A.V., Feneva D.M. (2018). Mercury in Tissues of Red Fox as Indicator of Environmental Pollution. Adv. Eng. Res..

