# Supplementary material for: Bioaccumulation of Heavy Metals (17 Elements) in the Liver and Kidneys of the Least Weasel (Mustela nivalis L.) from Agricultural Areas of Central Europe
Source: Toxics. 2026 Jan 27;14(2):118. doi: 10.3390/toxics14020118 (PMC12945121; doi:10.3390/toxics14020118)
Supplement: Supplementary file 1 [file toxics-14-00118-s001.zip › toxics-4065991-supplementary.pdf]

Supplementary Materials

Tables

Table S1. Concentrations of Fe, Zn, and Cu in liver and kidney tissue in mustelidae and canine predators.

Location	Species	Heavy metals concentrations (mg/kg $^{-1}$ wet weight)								
		Fe		*Ref.*	Zn		*Ref.*	Cu		*Ref.*
		L	*K*		*L*	*K*		*L*	*K*	
Croatia	*Martes martes*	–	–	–	–	–	–	10,03	4,94	[16]
Italia		–	–	–	99,12	–	[19]	22,37	–	[19]
Italia	*Martes foina*	–	–	–	71,87	–	[19]	21,08	–	[19]
Croatia		–	–	–	–	–	–	20,6[a]; 36,1[b]	3,19a; 9,02[b]	[15]
Spain	*Meles meles*	–	–	–	49,94	41,32	[23]	–	–	–
		–	–	–	40,25	–	[14]	24,97	–	[14]
Netherland		–	–	–	–	–	–	–	6,87	[138]
Croatia		–	–	–	–	–	–	15,20	4,69	[16]
Romania	*Vulpes vulpes*	208,14	–	[85]	31,31	16,62	[85]	17,70	–	[85]
Austria		165,76	–	[164]	–	–	–	23,31	–	[164]
Hungary		163,35	49,09	[165]	43,78	21,96	[82]	5,97	2,32	[82]
Poland		103,87	44,58	[84]	35,78	14,76	[84]	8,47	3,35	[84]
		205,48	80,38	[74]	48,88	25,98	[74]	7,69	3,62	[74]
Spain		–	–	–	35,73	–	[14]	20,08	–	[14]
		–	–	–	21,48	4,28	[31]	–	–	–
Croatia		–	–	–	–	–	–	15,60[a]; 27,30[b]	17,1[a]; 18,70[b]	[15]
Bulgaria		–	–	–	8,39	7,59	[86]	4,21	2,13	[86]
Serbia	*Canis aureus*	283,81	–	[83]	18,51	–	[83]	16,14	–	[83]
Croatia		249,0	–	[76]	25,3	–	[76]	14,50	–	[76]
Hungary		200,48	64,91	[165]	–	–	–	–	–	–
Romania		186,86	–	[85]	30,87	18,66	[85]	22,22	–	[85]
Bulgaria		–	–	–	17,74	15,51	[86]	15,71	8,53	[86]

[a] concentration measured in wild specimens, [b] specimens from urban environments; *L* - liver; *K* – kidney

Table S2. Concentrations of Mn, Se, and Cr in liver and kidney tissue in mustelidae and canine predators.

Location	Species	Heavy metals concentrations (mg/kg $^{-1}$ wet weight)								
		Mn		*Ref.*	Se		*Ref.*	Cr		*Ref.*
		L	*K*		*L*	*K*		*L*	*K*	
Spain	*Meles meles*	–	–	–	1,36	–	[14]	–	–	–
Romania	*Vulpes vulpes*	4,43	–	[85]	–	–	–	0,38	–	[85]
Spain		–	–	–	0,47	–	[14]	–	–	–
Poland		–	–	–	0,26	0,60	[91]	–	–	–
		–	–	–	0,24[a]	0,58[a]	[90]	–	–	–
		3,59	1,42	[74]	–	–	–	0,02	0,03	[74]
Slovakia		–	–	–	–	–	–	0,26	0,29	[120]
Serbia	*Canis aureus*	4,72	–	[83]	–	–	–	–	–	–
Croatia		3,01	–	[76]	0,32	–	[76]	–	–	–
Romania		3,85	–	[85]	–	–	–	0,13	–	[85]

concentration measured in specimens [a] from farms; *L* - liver; *K* - kidney

Table S3. Concentration of Mo and Co in liver and kidney tissue in canine predators.

Location	Species	Heavy metals concentrations (mg/kg $^{-1}$ wet weight)					
		Mo		*Ref.*	Co		*Ref.*
		L	*K*		*L*	*K*	
Hungary	*Vulpes vulpes*	–	–	–	0,449	0,618	[82]
Poland		0,27	0,13	[74]	–	–	–
		–	–	–	–	–	–
Romania		–	–	–	–	–	–
Bulgaria		–	–	–	0,512	0,464	[86]
Croatia	*Canis aureus*	0,258	–	[76]	0,023	–	[76]
Bulgaria		–	–	–	0,128	0,131	[115]
		–	–	–	1,38	0,26	[86]

L - liver; *K* – kidney

Table S4. Concentrations of Sn, Ti, and Ni in liver and kidney tissue in canine predators.

Location	Species	Heavy metals concentrations (mg/kg $^{-1}$ wet weight)								
		Sn		*Ref.*	Ti		*Ref.*	Ni		*Ref.*
		L	*K*		*L*	*K*		*L*	*K*	
Hungary	*Vulpes vulpes*	–	–	–	–	–	–	0,580	0,633	[82]
Poland					–	–	–	0,85	1,01	[84]
		0,328	0,267	[74]	–	–	–	0,007	0,007	[74]
Romania		–	–	–	–	–	–	0,36	–	[85]
Bulgaria		–	–	–	–	–	–	0,691	0,714	[86]
Croatia	*Canis aureus*		–		–	–	–	3,74	–	[83]
Bulgaria		–	–	–	–	–	–	0,078	0,128	[115]
		–	–	–	–	–	–	2,04	1,94	[86]
Romania		–	–	–	–	–	–	0,21	–	[85]

L - liver; *K* – kidney

Table S5. Concentrations of Hg, Pb, and Cd in liver and kidney tissue in mustelidae and canine predators.

Location	Species	Heavy metals concentrations (mg/kg $^{-1}$ w.w.)								
		Hg		*Ref.*	Pb		*Ref.*	Cd		*Ref.*
		L	*K*		*L*	*K*		*L*	*K*	
Italy	*Mustela nivalis*	–	–	–	0,08	–	[58]	–	–	–
Switzerland	*Mustela putorius*	–	1,04	[21]	–	0,42	[21]	–	0,11	[21]
Finland		0,39	0,66	[157]	–	–	–	–	–	–
Poland		0,04	0,08	[73]	–	–	–	–	–	–
Italy		–	–	–	0,45	–	[58]	0,08	–	[58]
Italy	*Martes foina*	0,11	–	–	0,33	–	[58]	0,96	–	[58]
		–	–	–	0,70	–	[19]	0,70	–	[19]
Croatia		0,029[a]; 0,021[b]	0,069[a]; 0,060[b];	[15]	0,130[a]; 0,349[b]	0,216[a]; 0,318[b]	[15]	0,155[a]; 0,161[b]	0,606[a]; 1,34[b]	[15]
Croatia	*Martes martes*	0,027	0,017	[16]	0,008	0,009	[16]	0,11	1,06	[16]
Poland		0,11	0,15	[73]	–	–	–	–	–	–
Italy		–	–	–	0,19	–	[19]	0,82	–	[19]
Netherland	*Meles meles*	–	–	–	–	0,62	[138]	–	14,37	[138]
Italy		0,18	–	[58]	0,40	–	[58]	0,67	–	[58]
Croatia		–	–	–	0,106	0,156	[16]	0,537	3,05	[16]
		0,037	0,084	[16]	0,197	0,190	[22]	0,395	3,046	[22]
Spain		0,025	–	[14]	0,128	–	[14]	0,0047	–	[14]
		0,21	–	[23]	0,086	0,113	[23]	1,31	1,91	[23]
Poland		0,17	0,24	[73]	–	–	–	–	–	–
Italy	*Vulpes vulpes*	0,044	–	[166]	0,24	–	[166]	0,165	–	[166]
		0,03	–	[58]	0,37	–	[58]	0,10	–	[58]
Croatia		0,009[a]; 0,025[b]	0,032[a]; 0,064[b]	[15]	–	–	–	0,125[b]	0,232[b]	–
		–	–	–	–	–	–	0,024a	0,054a	[15]
Hungary		–	–	–	0,468	0,662	[82]	0,139	0,206	[82]
		–	–	–	–	–	–	0,09	0,234	[165]
Poland		0,075	0,098	[84]	0,46	0,42	[84]	0,359	0,418	[84]
		–	–	–	0,179	0,112	[74]	0,016	0,061	[74]
Romania		–	–	–	0,52		[85]	0,97		[85]
Bulgaria		–	–	–	0,265	0,191	[86]	0,175	3,911	[86]
Slovakia		0,22	0,63	[120]	0,35	0,38	[120]	–	–	–
Spain		0,003	–	[14]	0,071	–	[14]	0,03	–	–
		–	–	–	0,225	0,01	[31]	–	–	–
Russia		0,123	0,240	[167]	–	–	–	–	–	–
Croatia	*Canis aureus*	0,045	–	[76]	0,439	–	[76]	–	–	–
Serbia		–	–	–	2,67	–	[83]	–	–	–
Bulgaria		–	–	–	1,91	1,01	[115]	–	–	–
		–	–	–	2,47	2,12	[86]	3,22	2,98	[86]
Romania		–	–	–	0,359	–	[85]	–	–	–

concentration measured [a] in wild specimens, [b] specimens from urban environment; *L* - liver; *K* – kidney

Table S6. Concentrations of V, Sb, and As in liver and kidney tissue in mustelidae and canine predators

**Location**	**Species**	**Heavy metals concentrations (mg/kg $^{-1}$ w.w.)**								
		**V**		*Ref.*	**Sb**		*Ref.*	**As**		*Ref.*
		L	*K*		*L*	*K*		*L*	*K*	
Croatia	*Martes foina*	–	–	–	–	–	–	0,017[a]; 0,014[b]	0,019[a]; 0,011[b]	[15]
Croatia	*Martes martes*	–	–	–	–	–	–	0,007	0,013	[16]
Croatia	*Meles meles*	–	–	–	–	–	–	0,33	0,012	[16]
Spain		–	–	–	–	–	–	0,039	0,020	[23]
Hungary	*Vulpes vulpes*	–	–	–	–	–	–	–	–	–
Spain		–	–	–	–	–	–	0,013	–	[14]
Croatia		–	–	–	–	–	–	0,013[a]; 0,014[b]	0,011[a]; 0,015[b]	[15]
Slovakia		–	–	–	–	–	–	0,38	0,30	[120]
Poland		0,055	0,075	[74]	–	–	–	–	–	–
Bulgaria		–	–	–	–	–	–	–	–	–
Bulgaria	*Canis aureus*	–	–	–	–	–	–	–	–	–
		–	–	–		–	–	–	–	–
Croatia		–	–	–	–	–	–	0,0046	–	[76]

concentration measured [a] in wild specimens, [b] specimens from urban environment; *L* - liver; *K* – kidney
